# Cbl interacts with multiple E2s *in vitro* and in cells

**DOI:** 10.1371/journal.pone.0216967

**Published:** 2019-05-23

**Authors:** Mariya S. Liyasova, Ke Ma, Donna Voeller, Philip E. Ryan, Jinqiu Chen, Rachel E. Klevit, Stanley Lipkowitz

**Affiliations:** 1 Women’s Malignancy Branch, Center for Cancer Research, National Cancer Institute, National Institutes of Health, Bethesda, Maryland, United States of America; 2 Collaborative Protein Technology Resource, Center for Cancer Research, National Cancer Institute, National Institutes of Health, Bethesda, Maryland, United States of America; 3 Department of Biochemistry, University of Washington, Seattle, Washington, United States of America; Hungarian Academy of Sciences, HUNGARY

## Abstract

Many receptor tyrosine kinases (RTKs, such as EGFR, MET) are negatively regulated by ubiquitination and degradation mediated by Cbl proteins, a family of RING finger (RF) ubiquitin ligases (E3s). Loss of Cbl protein function is associated with malignant transformation driven by increased RTK activity. RF E3s, such as the Cbl proteins, interact with a ubiquitin-conjugating enzyme (E2) to confer specificity to the ubiquitination process and direct the transfer of ubiquitin from the E2 to one or more lysines on the target proteins. Using *in vitro* E3 assays and yeast two-hybrid screens, we found that Ube2d, Ube2e families, Ube2n/2v1, and Ube2w catalyze autoubiquitination of the Cbl protein and Ube2d2, Ube2e1, and Ube 2n/2v1 catalyze Cbl-mediated substrate ubiquitination of the EGFR and SYK. Phosphorylation of the Cbl protein by by Src resulted in increased E3 activity compared to unphosphorylated cbl or Cbl containing a phosphomimetic Y371E mutation. Ubiquitin chain formation depended on the E2 tested with Cbl with Ube2d2 forming both K48 and K63 linked chains, Ube2n/2v1 forming only K63 linked chains, and Ube2w inducing monoubiquitination. In cells, the Ube2d family, Ube2e family, and Ube2n/2v1 contributed to EGFR ubiquitination. Our data suggest that multiple E2s can interact with Cbl and modulate its E3 activity *in vitro* and in cells.

## Introduction

Cbl proteins are a family of RF E3s that negatively regulate signaling by many tyrosine kinases (*e*.*g*., EGFR, Met, and Src) and tyrosine kinase-dependent pathways *(e*.*g*., T-cell receptor) [1–4]. The loss of Cbl E3 function can result in malignant transformation both in murine models and in human cancers due to hyperactivity of tyrosine kinases driven pathways [5, 6] There are three mammalian Cbl proteins: Cbl (*a*.*k*.*a*., c-Cbl, Cbl2, and RNF55), Cbl-b (*a*.*k*.*a*., RNF56), and Cbl-c (a.k.a., Cbl-3, Cbl-SL, and RNF57) [7]. Cbl proteins have a highly conserved N-terminus consisting of a tyrosine kinase binding (TKB) domain that binds to specific phosphorylated tyrosines on substrates, a catalytic RF domain, and an alpha helical linker region separating the TKB and RF domains [7]. The C-termini of the proteins are less highly conserved, although all three mammalian Cbl proteins have a proline rich (PR) region that mediates interaction with SH3-domain containing proteins. Cbl and Cbl-b have ubiquitin associated (UBA) domains at their C-termini that mediate homodimerization or ubiquitin binding, respectively [8–10]. The E3 activity of the Cbl proteins is negatively regulated by the N-terminus of the proteins and their activity is increased upon phosphorylation of a conserved tyrosine in the linker region [11, 12].

Ubiquitination occurs via a multistep reaction involving a ubiquitin-activating enzyme (E1), a ubiquitin-conjugating enzyme (E2), and a ubiquitin ligase (E3) [13]. Ubiquitination is initiated by the ATP dependent covalent attachment of the ubiquitin molecule to the active site cysteine of E1 [13–16]. There are two E1 enzymes in mammalian cells [13, 16]. The ubiquitin molecule is then transferred to the active site cysteine on a ubiquitin-conjugating enzyme (E2). There are around 40 E2s in mammalian cells [13–16]. Subsequently, the E2 interacts directly with an E3, which facilitates the transfer of a ubiquitin molecule to the specific substrate [15, 16]. There are several hundred E3s in mammalian cells [16]. The RF E3s, such as Cbl proteins, facilitate the transfer of ubiquitin from the E2 directly to one or more lysines on the target protein without a covalent E3-ubiquitin intermediate [17, 18]. E2 enzymes are essential for conjugation of ubiquitin and directly influence the type of ubiquitin chains formed on the substrates thereby influencing the fate of the substrate [14–16]. Crystallography studies demonstrated the interaction between Ube2l3 and Cbl [19]. Ube2l3 was shown to cooperate with Cbl and promote ubiquitination of EGFR, a well-studied Cbl RTK substrate [20]. However, more recent *in vitro* studies, although confirming the interaction between Cbl and Ube2l3, indicated that this E2-E3 complex might not be functional [21]. Members of the Ube2d family of E2s were also found to bind to Cbl and support its E3 activity *in vitro* [11, 21–23]. Moreover, RNAi mediated silencing of the Ube2d family in HeLa cells attenuated Cbl mediated ubiquitination of the activated EGFR [23]. Since at least two families of E2s were shown so far to interact with Cbl, we hypothesized that other, yet unidentified, E2s might cooperate with Cbl. Recently, it was established that multiple E2s may function with a specific RF E3 such as BRCA1 [24, 25]. In this work we investigated the spectrum of E2s that can interact with Cbl and support its E3 activity *in vitro* and in human cells.

## Material and methods

### Materials

Dulbecco’s modified Eagle’s medium (DMEM) and fetal bovine serum (FBS) were obtained from Invitrogen (Carlsbad, CA). Dulbecco’s phosphate buffered saline (DPBS) was purchased from Mediatech Inc. (Herndon, VA). LB broth (BLF-7030) was from KD Medical (Columbia, MD). Sodium orthovanadate was from Fisher Chemicals (Fairlawn, NJ). NP40 Cell Lysis Buffer (FNN0021) was from ThermoFisher Scientific (Waltham, MA). Recombinant human EGF was purchased from BD Biosciences, Inc. (San Jose, CA). Tissue culture plasticware and other laboratory consumables were purchased from commercial sources.

### Antibodies

Anti-ubiquitin (P4D1, sc-8017), anti-Cbl (C-15, sc-170), anti-Cbl-b (H-121, sc-1704), anti-Cbl-b (H454, sc-1705), anti-Cbl-b (G1, sc-8006), anti-Ube2d (C-6, sc-166278), anti-GST (sc-138), anti-Ube2e2 (sc-130052), anti-HSC70 (sc-7298) antibodies were obtained from Santa Cruz Biotechnology (Santa Cruz, CA). The anti-Ube2e1 (ab36980) antibody was from Abcam (Cambridge, MA). The anti-Ube2e3 (MABS17), anti-Syk (05–434), anti-Src (04–889), anti-ubiquitin K63-specific (05–1308), and K48-specific (05–1307) antibodies were obtained from EMD Millipore (Burlington, MA). The anti-Ube2w (HPA045161), anti-actin (A5316), and anti-FLAG (A8592) antibodies were obtained from MilliporeSigma (St. Louis, MO). The anti-EGFR antibody (199.12, MA5-13319) was purchased from ThermoFisher Scientific (Waltham, MA) and used for immunoprecipitation. The anti-EGFR (2232 and 4276) antibodies were obtained from Cell Signaling Technology (Danvers, MA) and used for immunoblotting. The anti-ubiquitin antibody Z0458 was obtained from DAKO (Carpinteria, CA).

### Yeast two-hybrid screening

Yeast two-hybrid screening was carried out using the N-terminal portion of Cbl (amino acids 2–469) as the bait. The Cbl cDNA was cloned into pGBKT7 bait vector (Clontech, Mountain View, CA) with Cbl fused to the GAL4 DNA-binding domain. Cbl-b (aa 2–461) and Cbl-c (aa 2–430) constructs in pGBKT7 vector were also generated. The E2 library constructs, in the pACT2 prey vector (Clontech), were kindly provided by Dr. Rachel Klevit. These constructs were previously used to identify E2s that interact with the BRCA1 RF [24]. Empty DNA-binding domain (DB) and empty activation domain (AD) vectors were used to control for nonspecific interactions of bait and prey, respectively. Using yeast transformation system 2 from Clontech, the yeast strain AH109 was co-transformed with respective bait and prey plasmids and positive transformants were grown either on media lacking leucine and tryptophan (-Leu -Trp) to identify yeasts that had received both plasmids or on media lacking histidine, leucine, and tryptophan supplemented with 5 mM 3-amino-1,2,4-triazole (3AT, MilliporeSigma, St. Louis, MO) (-His -Leu -Trp, +3AT) to identify interacting proteins. Yeasts were incubated for 2 weeks at 30°C and then colony formation was assessed. Cbl mutants (Y371E, C381A and Y371E/C381A), as well as Cblb (C373A) and Cbl-c (C351A) mutants were generated by QuikChange II Site-Directed Mutagenesis Kit (Stratagene, La Jolla, CA).

### Expression constructs

To generate GST-tagged Cbl, WT construct (aa 2–469) was cloned into pGEX-5x-1 or pGEX-6p-1 that were purchased from GE Healthcare Biosciences (Pittsburgh, PA). Site-directed mutagenesis using QuikChange Kit (Stratagene, La Jolla, CA) was performed to create GST-Cbl Y371E construct. Cbl-b (aa 2–483) and Cbl-c (aa 1–421) constructs were cloned into pGex2TK and pGEX-6p-1 vectors, respectively. Site-directed mutagenesis with QuikChange Kit was used to create GST-Cbl-b Y363E and GST-Cbl-c Y341E constructs. pET15b-Ube2d2 was provided by Dr. Allan Weissman. Plasmids for overexpression in HEK293T cells were purchased from OriGene (Rockville, MD): pCMV6 (PS10001), Ube2e1 (RC202106), Ube2e2 (RC204787), Ube2e3 (RC200274). All the constructs were confirmed by DNA sequencing.

### Production and purification of GST proteins

Expression vectors were transformed into Rosetta bacteria. Selected colonies were grown in LB broth with 100 μg/ml ampicillin for selection. Ten-ml overnight cultures of bacteria with GST-Cbl WT and GST-Cbl Y371E constructs were diluted with 90 ml of LB and incubated at 37°C for 1 h, followed by incubation at 16°C for 1 h and stimulation with 2 mM isopropyl β-D-thiogalactoside (IPTG; Sigma-Aldrich, St. Louis, MO) at 16°C overnight. Bacterial culture with the GST construct was diluted 1/10 with 90 ml of LB and cultured at 37°C for 4 h with 2 mM IPTG. Cell pellets were resuspended in 5 ml of lysis buffer (50 mM Tris, 1 mM EDTA, 1% Triton X-100, 5 mM dithiothreitol, pH 8), sonicated, and clarified by centrifugation. Then, 300 μl of a 50% slurry of glutathione-sepharose 4B from GE Healthcare Biosciences (Pittsburgh, PA) was added to the clarified lysate and incubated with rocking overnight at 4°C. Beads were pelleted by centrifugation and washed 5 times in lysis buffer and then 5 times in cold PBS. Beads were resuspended to 50% slurry in PBS for storage. Where indicated, the Cbl protein was cleaved from the GST protein using PreScission Protease as described by the supplier GE Healthcare Biosciences (Pittsburgh, PA).

### *In vitro* E3 assays

*In vitro* E3 assays were performed as previously described [12]. Briefly, 30 μl reactions contained 50 mM Tris-HCl pH7.5, 0.2 mM ATP, 0.5 mM MgCl_2_, 0.1 mM DTT, 1 mM phosphocreatine di(tris)salt (Sigma, St. Louis, MO), 15U creatine phosphokinase (EMD Biosciences, San Diego, CA), 50 ng (14 nM) purified recombinant rabbit ubiquitin-activating enzyme (E1; 662072, EMD Biosciences, San Diego, CA) and 0.5 μg ubiquitin (U100H; Boston Biochem, Cambridge, MA), K63R (UM-K63R), K48R (UM-K48R), or K0 ubiquitin (UB-NOK; Boston Biochem, Cambridge, MA). In the in vitro autoubiquitination screen, E2 enzyme combinations were added as indicated 1 μg (1–2.4 μM depending on the MW), along with 500 ng of either GST-tagged Cbl protein (~0.2 μM) coupled to glutathione-sepharose beads, GST-tagged Cbl (Y371E) protein coupled to glutathione-sepharose beads, or untagged Cbl WT protein cleaved from the glutathione sepharose beads. All of the E2 recombinant proteins were bought from either Life Sensors (Malvern, PA) or Boston Biochem (Cambridge, MA). The assays were performed at 30°C with constant shaking at 1000 RPM. General incubation time was 1 h unless otherwise stated. Reactions were stopped by addition of 2x Laemmli Sample Buffer (1610737, BioRad, Hercules, CA) supplemented with 0.75 M β-mercaptoethanol and boiling for 5 min. To precipitate GST-Cbl, a 10–15 μl aliquot was taken prior to stopping the reaction and diluted with 350 μl of Lysis buffer (10 mM TrisHCl pH 7.5, 150 mM NaCl, 5 mM EDTA, 1% Triton X-100, 10% glycerol) supplemented with 30 μl of glutathione agarose beads (50% slurry, sc-2009, Santa Cruz Biotechnology). Syk was immunoprecipitated from 15 μl aliquot with anti-Syk antibody and Protein A/G+ agarose beads (sc-2003, Santa Cruz Biotechnology, Dallas, Tx). Untagged Cbl constructs were immunoprecipitated using the anti-Cbl-b antibodies that cross react with all Cbl proteins (H454, sc-1705, Santa Cruz Biotechnology, Dallas, Tx) and EGFR was immunoprecipitated with an anti-GST antibody and Protein A/G+ agarose beads. IPs were incubated overnight with tumbling at 4°C, washed 4 times with lysis buffer, mixed with 30 μl of 2x Laemmli Sample Buffer and boiled for 7 min. Samples were analyzed with immunoblotting as described below.

Where indicated, Cbl proteins were phosphorylated by preincubation with 100 ng of active Src (14-326-D, EMD Millipore, Burlington, MA) or 500 ng of active Syk (14–314, EMD Millipore, Burlington, MA) in the E3 assay buffer without E2. Cbl proteins were phosphorylated by preincubation with 500 ng of active GST-EGFR cytoplasmic domain (40187, BPS Bioscience, San Diego, CA) in the phosphorylation buffer supplied with the GST-EGFR. Phosphorylated Cbl proteins were tested for phosphorylation by immunoblotting or used in the E3 assays with the addition of E2 as indicated in the results.

When samples were denatured prior to immunoprecipitation, a 15 μl aliquot was taken prior to stopping the reaction, added to 15 μl of 2x denaturation buffer (20 mm TRIS pH 7.4, 50 mM NaCl, 5 mM DTT, 1% SDS, 1 mM Sodium orthovanadate), and boiled for 6 minutes. The samples were then put on ice and brought to a total volume of 800 μl with NP40 cell lysis buffer (50 mM Tris, pH 7.4, 250 mM NaCl, 5 mM EDTA, 50 mM NaF, 1 mM Na_3_VO_4_, 1% Nonidet P40 (NP40), 0.02% NaN_3_), indicated antibody and Protein A/G+ agarose beads.

### siRNA transfections

HeLa cells (CCL2) were obtained from ATCC (Manassas, VA) and maintained in culture in DMEM supplemented with 10% FBS. Transfection was performed with Lipofectamine RNAiMAX Transfection Reagent (ThermoFisher Scientific, Waltham, MA). Cells were transfected for 24 hours and then trypsinized, pooled and replated to make the transfections equal across sets. The cells were further incubated for 24–48 h and then starved in DMEM medium without serum for 3 hours before stimulation with 25 ng/ml EGF. Immunoblotting and immunoprecipitation were done as described below. siRNAs for negative control (D-001810-10-20), Ube2e1 (L-008850-00-0005), Ube2e2 (L-031782-00-0005) and Ube2e3 (L-008845-00-0005) were purchased from Dharmacon (Lafayette, CO). siRNAs for Cbl (s2476), Cbl-b (s2479), Ube2d1 (s14573), Ube2d2 (s14574), Ube2d3 (s14579), Ube2d4 (s28419), Ube2n (120328), Ube2w (121838), and negative control (4390844) were purchased from ThermoFisher Scientific (Waltham, MA).

### Plasmid transfection to HEK293T cells

The human embryonic kidney (HEK293T) cells were obtained from ATCC and maintained in culture in DMEM supplemented with 10% FBS. Calcium phosphate (ProFection; Promega Corp., Madison, WI) was used for plasmid transfection according to the protocol of the manufacturer. Eighteen hours post transfection, medium on transfected cells was replaced with fresh media. At 48 hours post transfection, cells were harvested and lysed. Each experiment was repeated at least 3 times.

### Immunoblotting and immunoprecipitation from cellular lysates

To harvest proteins, cells were washed twice in ice-cold DPBS and then lysed in ice-cold NP40 cell lysis buffer (50 mM Tris, pH 7.4, 250 mM NaCl, 5 mM EDTA, 50 mM NaF, 1 mM Na_3_VO_4_, 1% Nonidet P40 (NP40), 0.02% NaN_3_) supplemented with 1 mM sodium orthovanadate (Na_3_VO_4_) and protease inhibitors (Complete tabs, MilliporeSigma, St. Louis, MO). The lysates were cleared of debris by centrifugation at 13,000 g for 10 min at 4°C. The supernatants were collected and protein concentrations were determined using a BioRad protein assay (BioRad, Hercules, CA). For immunoblotting, lysates were boiled in Hercules, CA). For immunoblotting, lysates were boiled in SDS-PAGE protein loading buffer for 7 min. For immunoprecipitation, transfected HeLa or HEK293T lysates containing 1 mg protein were incubated with anti-EGFR (199.12) or anti-Cbl (C-15) antibody and Protein A/G+ agarose beads (Santa Cruz Biotechnology, Santa Cruz, CA) overnight at 4°C with tumbling. Immune complexes were washed five times in cold lysis buffer, resuspended in 2x Laemmli Sample Buffer for 7 min. For immunoprecipitation, transfected HeLa or HEK293T lysates containing 1 mg protein were incubated with anti-EGFR (199.12) or anti-Cbl (C-15) antibody and Protein A/G+ agarose beads (Santa Cruz Biotechnology, Santa Cruz, CA) overnight at 4°C with tumbling. Immune complexes were washed five times in cold lysis buffer, resuspended in Hercules, CA). For immunoblotting, lysates were boiled in SDS-PAGE protein loading buffer for 7 min. For immunoprecipitation, transfected HeLa or HEK293T lysates containing 1 mg protein were incubated with anti-EGFR (199.12) or anti-Cbl (C-15) antibody and Protein A/G+ agarose beads (Santa Cruz Biotechnology, Santa Cruz, CA) overnight at 4°C with tumbling. Immune complexes were washed five times in cold lysis buffer, resuspended in 2x Laemmli Sample Buffer and boiled for 7 min. The proteins were resolved by SDS-PAGE, transferred to nitrocellulose membranes (Protran BA85; Whatman, Sanford, MA), and incubated with primary antibody overnight at 4°C. Horseradish peroxidase linked donkey anti-rabbit IgG (NA934V; GE Healthcare, Piscataway, NJ), horseradish peroxidase linked donkey anti-mouse IgG (NA931: GE Healthcare, Piscataway, NJ), goat anti-rabbit IgG HRP conjugate (1721019, Biorad, Hercules, CA), or goat anti-mouse IgG HRP conjugate (1721011, Biorad, Hercules, CA) were used with SuperSignal (Pierce Biotechnology Inc., Rockford, IL) to visualize target proteins, using either a film processor or Li-cor Odyssey Imager (Li-Cor Biotechnology, Lincoln, NE). Alternatively, the KwikQuant Western Blot Detection Kit (R1004) and KwickQuant Imager (D1001) from Kindle Biosciences, LLC (Greenwich, CT) were used to detect proteins on the nitrocellulose membrane. Each experiment was repeated at least 3 times. Densitometric analysis of immunoblot band intensities was performed using Adobe Photoshop software version CC 2017 (Adobe Systems Inc., U.S.A) or Image Studio Software (Li-Cor, Biotechnology Inc. Rockford, IL).

### Simple western

HeLa cells were harvested and lysed with lysis buffer as above. Approximately 30 to 72 ng of protein was used per sample, and experiments were performed using a Peggy Sue instrument (ProteinSimple, San Jose, CA), as previously described [26]. Ube2w expression level was detected using an anti-Ube2w primary antibody with an HRP-conjugated goat anti-rabbit secondary antibody (Jackson ImmunoResearch Laboratories, West Grove, PA). The digital images were analyzed and quantified with Compass software (ProteinSimple, San Jose, CA). Total protein was measured as loading control.

### Real-time PCR

To assess the expression levels and knockdown efficiency of Ube2d proteins in HeLa cells, RT-QPCR was performed. First, RNA was isolated from transfected HeLa cells using RNeasy Mini Kit (74104, Qiagen) according to manufacturer’s instructions. RNA concentrations were measured with NanoDrop 2000 (ThermoFisher). 400 ng of total RNA was then used to generate cDNA with QuantiTect Reverse Transcription Kit (205313, Qiagen). Twenty microliters of the RT reaction were diluted to 150 μl with RNase-free water and used in RT-PCR at 5 μl per reaction in the total volume of 15 μl containing PowerUp SYBR Green Master Mix (A25742, ThermoFisher) and specific primers. QuantiTect primers to human beta actin (QT00095431), Ube2d1 (QT00022337), Ube2d2 (QT01009869), Ube2d3 (QT00076545), Ube2d4 (QT00005593) were purchased from Qiagen. RT-QPCR was performed with ViiA 7 Real-Time PCR System (Applied Biosystems).

## Results

### Ube2d family members mediate the autoubiquitination of Cbl proteins *in vitro*

**Fig 1:**
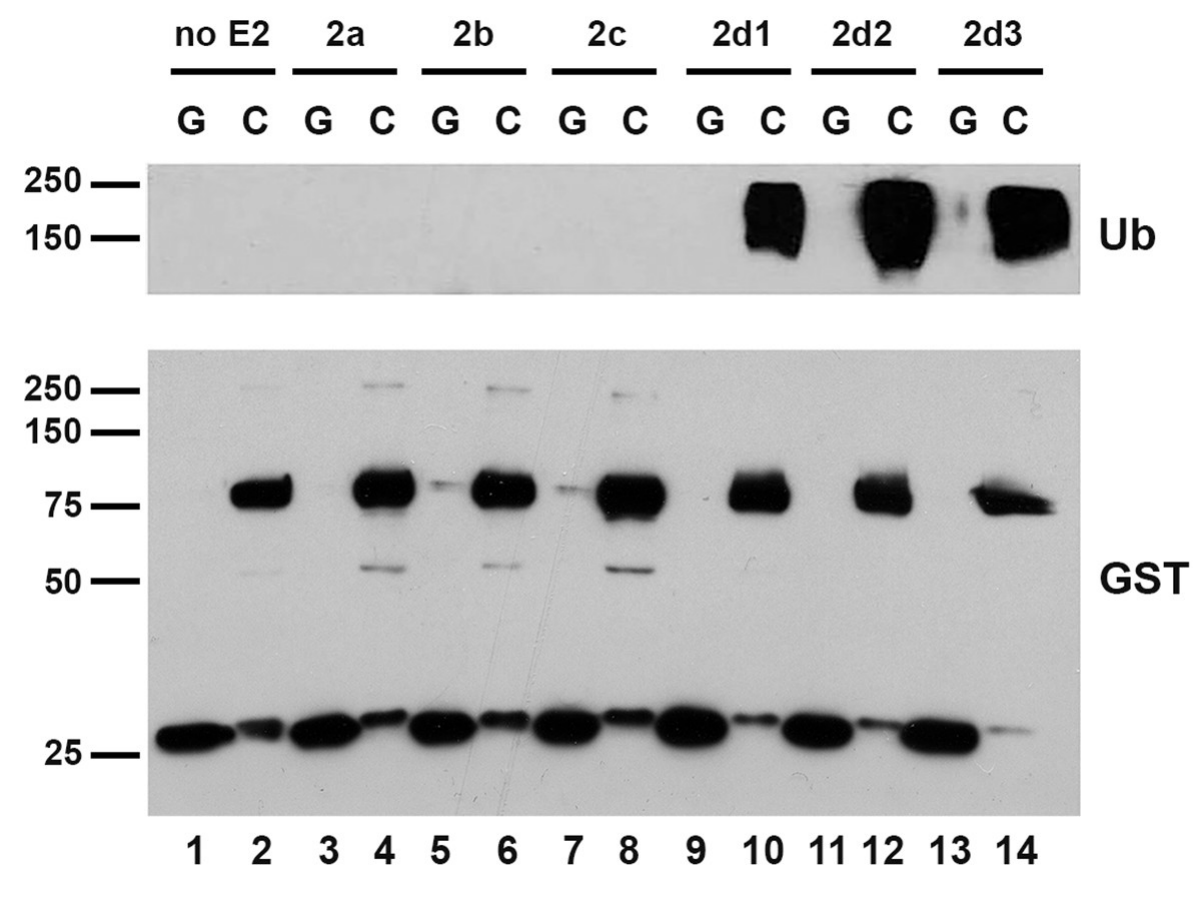
Ube2d family members mediate autoubiquitination of Cbl *in vitro*. In total 28 mammalian E2s were tested in an *in vitro* E3 assay for their ability to mediate autoubiquitination by GST-Cbl (aa 2–469) with activating Y371E mutation (C). A representative panel is showing that Ube2d1, Ube2d2 and Ube2d3 mediate autoubiquitination of Cbl while Ube2a, Ube2b, and Ube2c do not. GST (G) protein was used as a negative control. Reactions were immunoblotted and probed for ubiquitin (top panel). Parallel blots were probed for GST (bottom panel) demonstrating equal GST-Cbl and GST proteins loading. MW in kDa is shown to the left of the panels.

Cbl proteins are RF domain-containing ubiquitin ligases involved in many signal transduction processes [2]. To investigate which E2s can function with the Cbl proteins to promote ubiquitin chain formation, *in vitro* autoubiquitination E3 assays were performed using recombinant E1 (Ube1), one of 28 recombinant E2s (Table 1), bacterially expressed GST-Cbl protein, and ubiquitin. In our *in vitro* experiments, we used truncated GST-Cbl proteins containing the N-terminal TKB domain and the RF (aa 2–469). To enhance the interaction with the E2s, a phospho-mimetic Y371E mutation was introduced into the Cbl proteins as this has been shown to enhance E3 activity [11, 12]. GST protein was used as a negative control. E3 activity was measured by a gel-based assay assessing autoubiquitination using an anti-ubiquitin antibody (Fig 1 and S1 Fig). Only Ube2d1, Ube2d2 and Ube2d3 were found to mediate autoubiquitination of Cbl. A representative sample of assays is shown in Fig 1 and the results are summarized in Table 1 and shown for all assays in S1A Fig. The screen was also performed for Cbl-b and Cbl-c. Again, only Ube2d1, Ube2d2 and Ube2d3 were found to mediate self-ubiquitination of Cbl-b and only Ube2d2 and Ube2d3 supported Cbl-c activity (S1B Fig and S1C Fig). The E2, Ube2l3, which has been co-crystalized with the RF of Cbl did not support E3 activity of Cbl (S1A Fig) [19]. Ube2n combined with Ube2v1 resulted in formation of free polyubiquitin chains with GST protein alone and therefore the result was indeterminant in this assay (S1 Fig). We will address the activity of Ube2n/2v1 further below. We did not see evidence for monoubiquitination with any E2 in either the ubiquitin or GST immunoblots (see discussion below). Thus, when using *in vitro* autoubiquitination assays only the Ube2d family was found to support E3 activity of Cbl proteins.

**Table 1:** E2s tested for activity with Cbl proteins *in vitro*.

E2	Cbl Y371E^1^	Cbl-b Y363E^2^	Cbl-c Y341E^3^
**Ube2a**	**-**	**-**	**-**
**Ube2b**	**-**	**-**	**-**
**Ube2c**	**-**	**-**	**-**
**Ube2d1**	**+**	**+**	**-**
**Ube2d2**	**+**	**+**	**+**
**Ube2d3**	**+**	**+**	**+**
**Ube2d4**	**-**	**-**	**-**
**Ube2e1**	**-**	**-**	**-**
**Ube2e2**	**-**	**-**	**-**
**Ube2e3**	**-**	**-**	**-**
**Ube2g1**	**-**	**-**	**-**
**Ube2g2**	**-**	**-**	**-**
**Ube2h**	**-**	**-**	**-**
**Ube2j1**	**-**	**-**	**-**
**Ube2k**	**-**	**-**	**-**
**Ube2l3**	**-**	**-**	**-**
**Ube2l6**	**-**	**-**	**-**
**Ube2n**	**-**	**-**	**-**
**Ube2q2**	**-**	**-**	**-**
**Ube2r1**	**-**	**-**	**-**
**Ube2r2**	**-**	**-**	**-**
**Ube2s**	**-**	**-**	**-**
**Ube2t**	**-**	**-**	**-**
**Ube2u**	**-**	**-**	**-**
**Ube2n/Ube2v1**	**-**	**-**	**-**
**Ube2v2**	**-**	**-**	**-**
**Ube2w**	**-**	**-**	**-**
**Ube2z**	**-**	**-**	**-**

All the E2s were tested in an *in vitro* E3 assay using activated Cbl (Y371E), Cbl-b (Y363E) and Cbl-c (Y341E) proteins. +, E2 supported E3 activity;–, E2 did not support E3 activity.

^1^GST-Cbl (aa 2–469) with activating Y371E mutation

^2^GST-Cbl-b (aa 2–483) with activating Y363E mutation

^3^GST-Cbl-c (aa 1–421) with activating Y341E mutation

### Identification of interactions between Cbl RF domain and ubiquitin-conjugating enzymes

**Fig 2:**
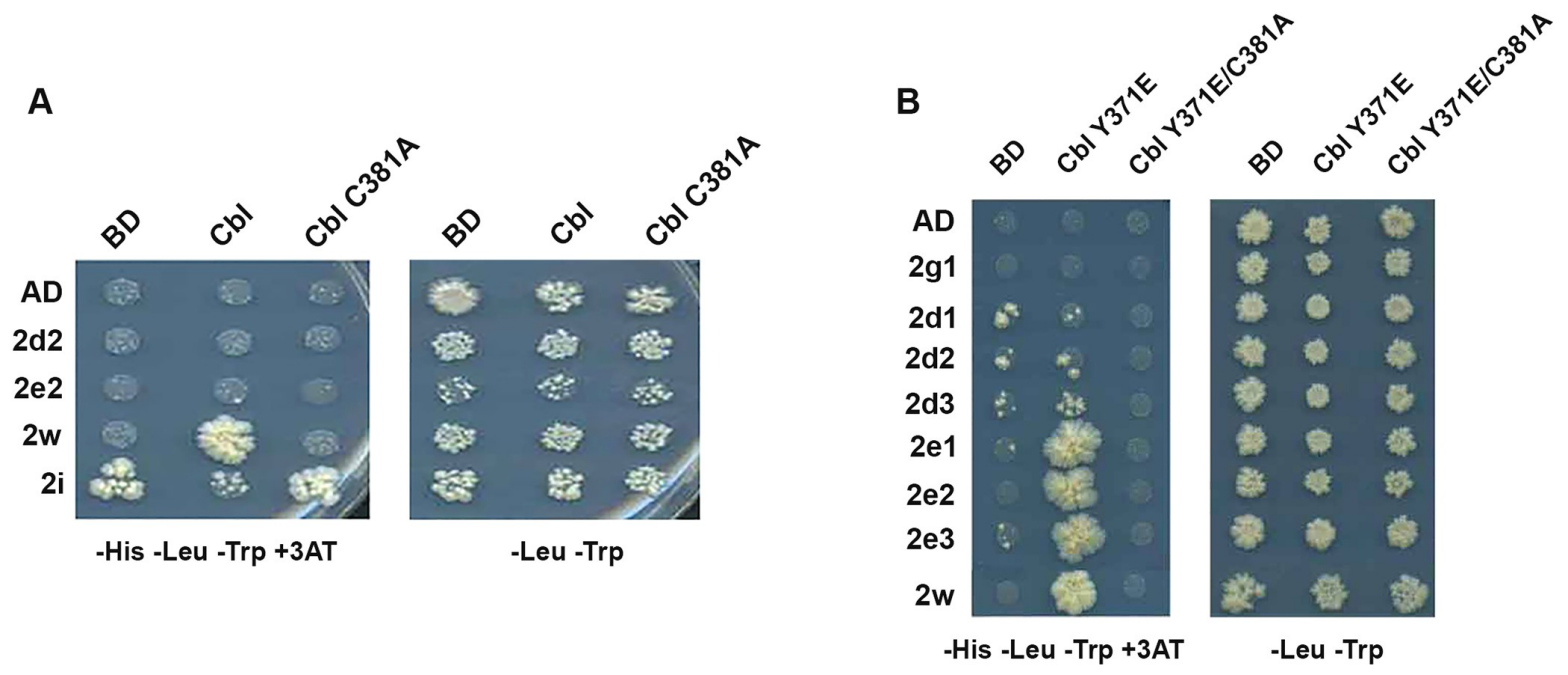
E2s interacting with intact RING finger domain of Cbl. (A) Representative yeast colonies transformed with wild-type Cbl or RF Mutant Cbl C381A as the bait and with the indicated E2s as prey. Empty DNA-binding domain (DB) and empty activation domain (AD) vectors were used as negative controls for bait and prey, respectively. (B) Representative yeast colonies transformed with activated Cbl Y371E or activated RF mutant Cbl Y371E/C381A as the bait with the indicated E2s as prey. Ube2e family members and Ube2w interact with activated Cbl Y371E but not with activated RF mutant Cbl Y371E/C381A. For both A and B, the left panel shows growth of the yeast on -His, -Leu, -Trp, +3AT interaction selective media and the right panel shows grow on -Leu, -Trp media to confirm that the yeast received both bait and prey plasmids.

To identify additional E2s interacting with Cbl, we then performed a directed yeast two-hybrid screen. For the screen, we used Cbl as bait with 29 human E2s, including human E2 variants, as prey (S2A Fig and Table 2). To validate the screen, BRCA1-BARD1 fusion constructs BC112-F-BD115 and BC112-F-BD115 I26A (I*A) were used with Ube2d proteins as positive and negative controls, respectively. In addition, selected E2s were tested for the interaction with Cbl-b and Cbl-c constructs (S2B Fig and S2C Fig, respectively). In the presence of wild-type Cbl, only co-transformation with Ube2w resulted in growth on the interaction selective media (-His, -Leu, -Trp, +3AT) that was significantly greater than the background (Fig 2A and S2A Fig). It has been shown that RF domain C381 is crucial for Cbl E3 activity both *in vitro* and *in vivo* [27]. In our screen, when Cbl C381A was co-transformed with Ube2w, no growth over background was observed on the interaction selective media, thus confirming the RF dependence of this interaction (Fig 2A). The same result was obtained when either Cbl-b or Cbl-c was studied: Ube2w interacted with both proteins in a RF-dependent manner (S2B Fig and S2C Fig). The E2 Ube2i resulted in growth on the interaction selective media when co-transformed with the empty BD vector and was thus not indicative of an interaction with Cbl (Fig 2A). Interestingly, none of the Ube2d E2s resulted in increased growth when co-transformed with Cbl on the interaction selective media (Fig 2A and S2 Fig). Similarly, Ube2l3 which has been co-crystallized with Cbl [19], did not support growth of the yeast when co-transformed with Cbl (S2 Fig). Since the yeast two-hybrid system requires a relatively stable interaction of bait and prey protein, it may not be able to detect the transient or low affinity interactions of E2 and E3 proteins. Published data suggest that phosphorylation of Cbl on the linker tyrosine Y371 increases the binding affinity for Ube2d2 [28]. In addition, *in vitro* assays have demonstrated greater activity when a phospho-mimetic Y to E mutation is introduced at this site [11]. We hypothesized that screening with the phospho-mimetic activated form of Cbl may reveal weaker interactions which could not be detected in our previous experiments. For this purpose, activated Cbl (Y371E) was then used to screen all 29 E2s (S3 Fig). Ube2w again supported growth on the interaction selective media (Fig 2B and S3 Fig). In addition, screening with the activated Cbl protein resulted in growth with Ube2e1, Ube2e2 and Ube2e3 (Fig 2B and S3 Fig). Furthermore, Ube2e1, Ube2e2, Ube2e3 and Ube2w did not support growth on the selective media when co-transformed with a Cbl bait that had both the activating mutation in the linker region and a RF inactivating mutation (Cbl Y371E/C381A). Ube2u interacted with activated Cbl Y371E, WT Cbl-b and Cbl-c, but these interactions were not dependent on RF (S2 Fig and S3 Fig) and thus were not studied further. Ube2v3 interacted with Cbl Y371E, but this result was not consistent. To conclude, the results from yeast two-hybrid screen suggest that all three Ube2e family members and Ube2w interact with Cbl proteins in a RF dependent manner (Fig 2B).

**Table 2:** E2s tested for interaction with Cbl in yeast two-hybrid screen.

E2	Cbl WT	Cbl Y371E	Cbl C381A	BD vector
**Ube2b**	**-**	**-**	**-**	**-**
**Ube2c**	**-**	**-**	**-**	**-**
**Ube2d1**	**-**	**-**	**-**	**-**
**Ube2d2**	**-**	**-**	**-**	**-**
**Ube2d3**	**-**	**-**	**-**	**-**
**Ube2e1**	**-**	**+**	**-**	**-**
**Ube2e2**	**-**	**+**	**-**	**-**
**Ube2e3**	**-**	**+**	**-**	**-**
**Ube2f**	**-**	**-**	**-**	**-**
**Ube2g1**	**-**	**-**	**-**	**-**
**Ube2h**	**-**	**-**	**-**	**-**
**Ube2i**	**+**	**+**	**+**	**+**
**Ube2k**	**-**	**-**	**-**	**-**
**Ube2l3**	**-**	**-**	**-**	**-**
**Ube2l6**	**-**	**-**	**-**	**-**
**Ube2m**	**-**	**-**	**-**	**-**
**Ube2n**	**-**	**-**	**-**	**-**
**Ube2q2**	**-**	**-**	**-**	**-**
**Ube2r1**	**-**	**-**	**-**	**-**
**Ube2s**	**-**	**-**	**-**	**-**
**Ube2t**	**-**	**-**	**-**	**-**
**Ube2u**	**-**	**+**	**+**	**-**
**Ube2v1**	**-**	**-**	**-**	**-**
**Ube2v2**	**-**	**-**	**-**	**-**
**Ube2v3**	**-**	**-**	**-**	**-**
**Uev1b**	**-**	**-**	**-**	**-**
**Ube2w**	**+**	**+**	**-**	**-**
**TSG101**	**-**	**-**	**-**	**-**
**FT1**	**-**	**-**	**-**	**-**

All the E2s were tested in yeast two-hybrid screening using wild-type Cbl (Cbl WT), Cbl mutant (Y371E) or Cbl RING finger mutant (C381A). +, growth present on selective medium;–, growth absent. The Ube2i is a self-activating vector showing positive interactions with all bait vectors.

### Phosphorylation of Cbl enhances autoubiquitination to a greater degree than the Y371E phosphomimetic mutation *in vitro*

**Fig 3:**
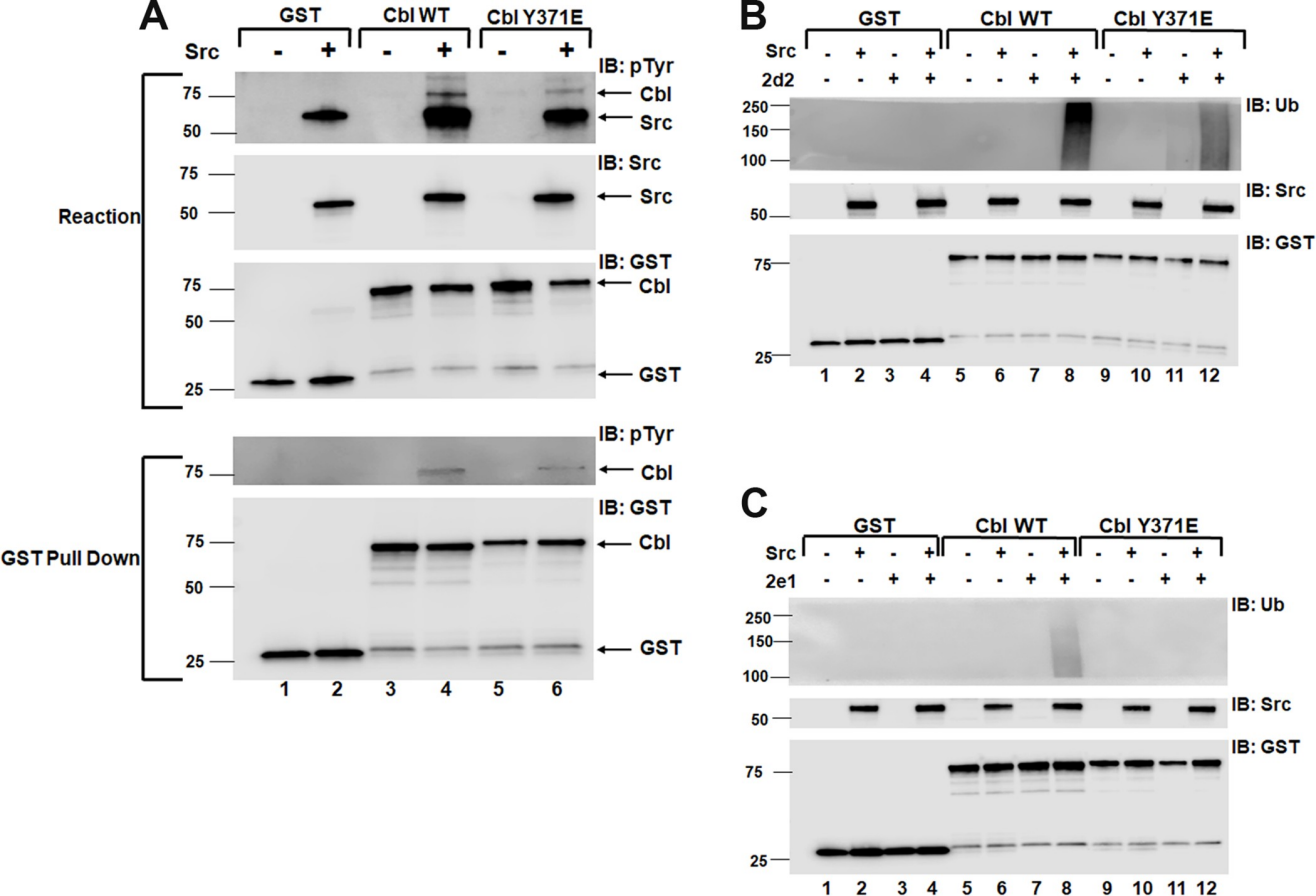
Phosphorylation of wild-type Cbl enhances autoubiquitination to a greater degree than the Y371E phosphomimetic mutation *in vitro*. (A) GST, wild-type GST-Cbl (Cbl WT) or Y371E mutant GST-Cbl (Cbl Y371E) were incubated in the E3 assay buffer lacking E2 for 60 min at 30°C. An aliquot of the reaction mixture was immunoblotted for phosphotyrosine (pTyr), Src, and Cbl (using the anti-GST antibody) as indicated. GST-Cbl was precipitated from a second aliquot of the reaction using GSH-agarose and again immunoblotted for phosphotyrosine or Cbl (using the anti-GST antibody). Aliquots of the phosphorylation reaction were tested for E3 activity with or without Ube2d2 (B) or Ube2e1 (C) as described in the methods. The E3 autoubiquitination assays were immunoblotted for ubiquitin (Ub), Src, or Cbl (using the anti-GST antibody) as indicated.

The yeast two-hybrid screen identified several E2s (the Ube2e family and Ube2w) that were not active in the initial assay-based screen shown in Fig 1 and S1 Fig. Prior work has shown that phosphorylation of Cbl proteins on a conserved linker tyrosine enhances E3 activity of the proteins and that a phosphomimetic Y to E mutation of this conserved tyrosine can also enhance E3 activity of the Cbl proteins [11, 12, 28]. However, it is possible that the more physiologic phosphorylation of the linker tyrosine results in higher activity than the phosphomimetic Y to E mutation, and possibly accounting for the lack of activity in our initial screen shown in Fig 1 and S1 Fig. To test this, we preincubated the wild-type or Y371E GST-Cbl protein with Src in the E3 reaction buffer without E2 to allow phosphorylation, and then tested the phosphorylated Cbl protein in an E3 autoubiqutination assay (Fig 3). Incubation of either wild-type Cbl or Y371E Cbl with Src resulted in tyrosine phosphorylation of Cbl but the wild-type Cbl was more heavily phosphorylated than the Y371E in both the reaction mixture (Fig 3A, top panel 1, compare lane 4 to lane 6) and the Cbl protein pull down (Fig 3A, bottom panel 4, compare lane 4 to lane 6).

Aliquots of the GST, GST-Cbl, or GST-Cbl Y371E proteins incubated in the presence or absence of Src shown in Fig 3A were used to test E3 activity (as measured by autoubiquitination) of the GST or GST-Cbl proteins with either Ube2d2 or Ube2e1 (Fig 3B and 3C, respectively). When incubated with Ube2d2, phosphorylated wild-type GST-Cbl had stronger E3 activity when compared to the unphosphorylated Y371E mutant indicating that phosphorylation results in a more active Cbl protein than the phosphomimetic Y371E mutation (Fig 3B compare lane 8 to lane 11). Under these reaction conditions, autoubiquitination was not seen with unphosphorylated wild-type GST-Cbl (Fig 3B, lane 7). Phosphorylation of the Y371E GST-Cbl increased E3 activity compared to unphosphorylated Y371E GST-Cbl, but not to the levels seen with phosphorylated wild-type GST-Cbl (Fig 3B, compare lane 12 to lanes 11 and 8 respectively). GST proteins in the presence or absence of Src did not result in any E3 activity (Fig 3B, lanes 1–4).

The E3 activities of the phosphorylated or unphosphorylated GST-Cbl proteins were also tested with Ube2e1. E3 activity was only seen with phosphorylated, wild-type GST-Cbl (Fig 3C, lane 8). The E3 activity of the phosphorylated wild-type GST-Cbl appeared greater when incubated with Ube2d2 compared to Ube2e1 (compare lane 8 in Fig 3B to lane 8 in Fig 3C). The assays in Fig 3B and 3C were done with equal aliquots of the same phosphorylated Cbl proteins but run on separate gels. To confirm this observation, the reactions were repeated with or without Src included in the E3 assays comparing Ube2d2 to Ube 2e1, 2e2, or 2e3. GST-Cbl had weak activity when incubated with Ube2d2 and this was dramatically increased when Src was included in the reaction (S4A Fig, compare lane 2 to lane 4). In the absence of Src, no activity was seen when GST-Cbl was incubated with Ube2e1, 2e2, or 2e3 (S4A Fig, lane 5). In the presence of Src, weak E3 activity compared to that with Ube2d2 could be detected when Cbl was incubated with Ube2e1, Ube2e2 and Ube2e3 (S4A Fig, lane 7). To test whether Ube2e proteins affect Ube2d2-mediated ubiquitination of Cbl, we modified the reactions to include a 20-min incubation of Cbl with three Ube2e family members individually as a first step, followed by addition of Ube2d2 for an additional 20 min. We found that the combination of Ube2e1, Ube2e2 or Ube2e3 with Ube2d2 resulted in decreased E3 activity of Cbl (S4A Fig) in the absence of Src (S4A Fig, compare Ube2d2 alone in lane 2 to Ube2e/Ube2d2 in lane 6) and in the presence of Src (S4A Fig, compare Ube2d2 alone in lane 4 to Ube2e/Ube2d2 in lane 8). These data suggest that three Ube2e family members act as weak E2s for Cbl and reduce Ube2d2-mediated ubiquitination of Cbl *in vitro* presumably by competing for E1 or Cbl with Ube2d2.

Taken together, the data in Fig 3 suggest that tyrosine phosphorylation of wild-type GST-Cbl results in greater activation of E3 activity than the phosphomimetic Y371E mutation and this could account for the lack of activity of the Ube2e family seen in the initial screen in Fig 1 and S1 Fig. Thus, we re-screened a panel of 12 E2s with E3 assays containing WT GST-Cbl coincubated with Src (Table 3). This screen confirmed the finding from the initial E3 assay screen that Ube2d1, Ube2d2 and Ube2d3 support E3 activity of Cbl in the presence of Src (S4B Fig, lanes 4, 6 and 8). Moreover, Ube2n combined with Ube2v1 also supported GST-Cbl E3 activity (S4B Fig, lane 10). In addition, we observed weak E3 activity in the presence of Ube2e family members as already demonstrated in Fig 3 and S4A Fig. Ube2w did not show activity in this screen incorporating Src (see below).

**Table 3:** E2s tested for activity with WT Cbl protein in the presence of Src *in vitro*.

E2	WT GST-Cbl +Src
**Ube2c**	**-**
**Ube2d1**	**+**
**Ube2d2**	**+**
**Ube2d3**	**+**
**Ube2e1**	**+**
**Ube2e2**	**+**
**Ube2e3**	**+**
**Ube2h**	**-**
**Ube2l3**	**-**
**Ube2r1**	**-**
**Ube2n/Ube2v1**	**+**
**Ube2w**	**-**

All the E2s were tested in an *in vitro* E3 assay using WT GST-Cbl (aa 2–469) in the presence of active full-length Src. +, E2 supported E3 activity;–, E2 did not support E3 activity.

### Multiple E2s collaborate with Cbl to ubiquitinate EGFR or Syk *in vitro*

**Fig 4:**
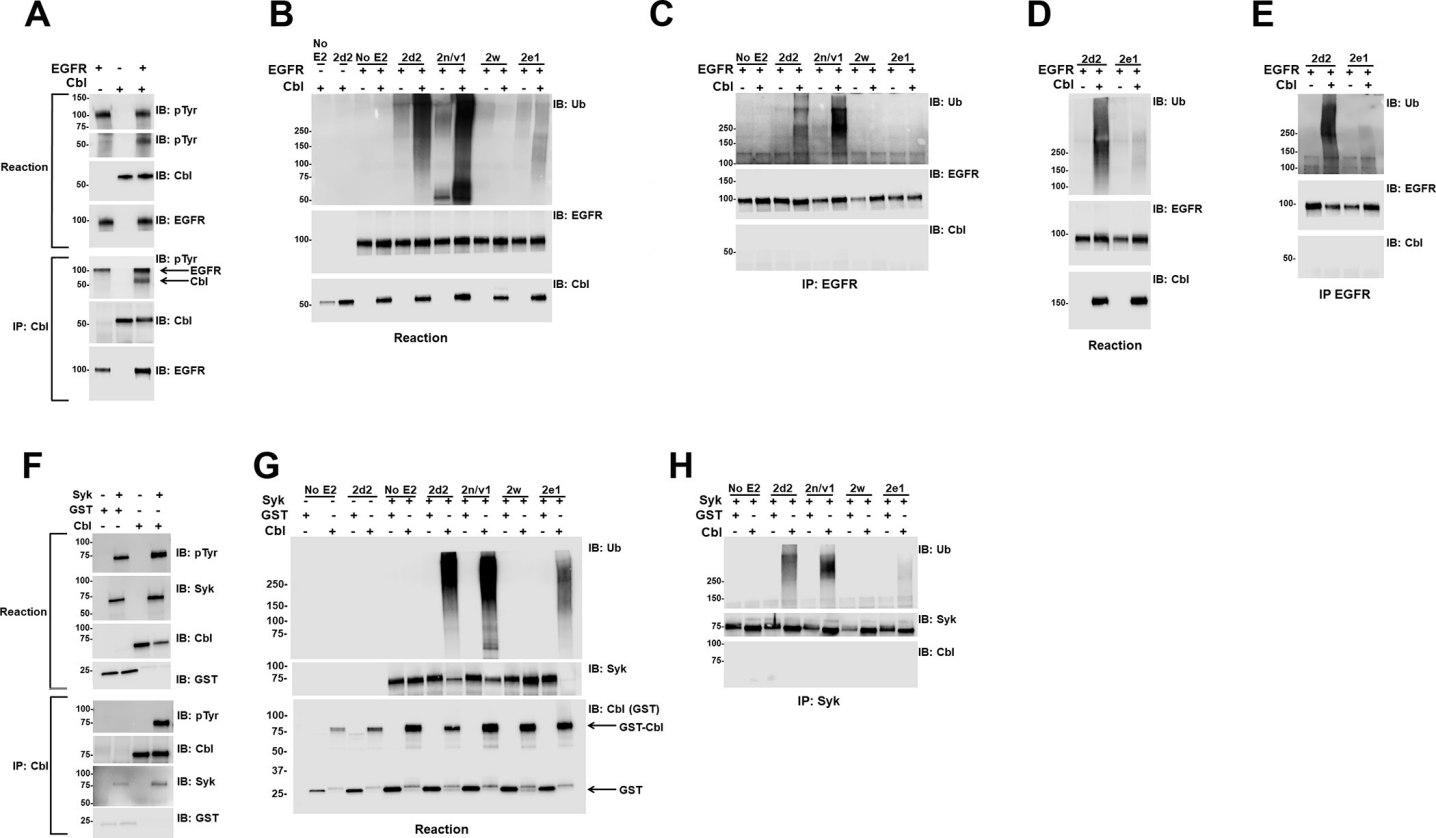
Phosphorylated Cbl mediates substrate ubiquitination *in vitro*. (A) Untagged Cbl N1/2, GST-EGFR, or the combination were incubated in the kinase assay buffer without E2 for 60 min at 30^o^ C to allow phosphorylation of Cbl. In the top panels, an aliquot of the reaction mixture was immunoblotted for phosphotyrosine (pTyr), EGFR (using anti-EGFR antibody), and Cbl (using the Cbl-b G1 N terminal antibody) as indicated. Cbl was precipitated from a second aliquot of the reaction using anti-Cbl-b antibody (H454) and again immunoblotted for phosphotyrosine, Cbl, and EGFR. (B) Aliquots of the phosphorylation reaction in A were tested for E3 activity with or without the E2s as indicated and probed for Ubiquitin, EGFR, and Cbl. (C) An aliquot of the ubiquitination reaction in B was denatured (as described in methods) and the EGFR was immunoprecipitated. The precipitate was probed for Ubiquitin, EGFR and Cbl as indicated. (D) The reactions in B were repeated using an increased amount of Ube2e1 and probed as indicated. (E) An aliquot of the ubiquitination reaction in D was denatured, EGFR was immunoprecipitated, and the precipitate was probed for Ubiquitin, EGFR and Cbl as indicated. (F) GST-Cbl N1/2, Syk, or the combination were incubated in the E3 assay buffer lacking E2 for 60 min at 30°C to allow phosphorylation of Cbl. In the top four panels, an aliquot of the reaction mixture was immunoblotted for phosphotyrosine (pTyr), Syk, Cbl, and GST as indicated. To assess Cbl phosphorylation, an aliquot of the phosphorylation reaction was denatured (as described in the methods). Cbl was immunoprecipitated using anti-Cbl-b antibody (H454), and again immunoblotted for phosphotyrosine, Syk, Cbl and GST (shown in panels 5–8). (G) Aliquots of the phosphorylation reaction in F were tested for E3 activity with or without the E2s as indicated and probed for Ubiquitin, Syk, and Cbl (using anti-GST antibody). (H) An aliquot of the ubiquitination reaction in G was denatured (as described in methods), Syk was immunoprecipitated and the precipitate was probed for Ubiquitin, Syk and Cbl as indicated.

The reactions described in Fig 1 above are primarily measuring autoubiquitination of the Cbl proteins. We next tested the ability of Cbl to ubiquitinate known substrates *in vitro*. Cbl-mediated ubiquitination of the activated EGFR in cells results in down regulation of the EGFR [20, 22, 29]. To test Cbl-mediated ubiquitination of the EGFR, we first preincubated recombinant untagged Cbl N1/2 with recombinant GST-EGFR containing the cytosolic domain of the EGFR in the phosphorylation buffer included with the GST-EGFR (Fig 4). Incubation of the Cbl protein and the GST-EGFR resulted in phosphotyrosine bands that comigrated with EGFR and Cbl (Fig 4A, top and 2^nd^ panel, respectively). The filter was cut and phosphotyrosine probed on the top two panels separately as the EGFR signal was much stronger and obscured the Cbl signal when run on the same panel. To confirm that the Cbl protein was phosphorylated, the Cbl protein was immunoprecipitated from the phosphorylation reaction, and immunoblotted for phosphotyrosine (Fig 4A, 5^th^ panel). When Cbl and EGFR are incubated together, a clear phosphotyrosine band is seen at the size of both Cbl and the EGFR. The EGFR was non-specifically immunoprecipitated by the anti-Cbl antibody and was phosphorylated in the presence or absence of Cbl (Fig 4A, 5^th^ panel, lane 1 and 3). Aliquots of the Cbl (+/- GST-EGFR) proteins from the phosphorylation step were used in E3 assays as shown in Fig 4B. In the absence of phosphorylation, no Cbl-mediated ubiquitination was observed (Fig 4B, lane 2). When phosphorylated Cbl was used, ubiquitination was seen with Ube2d2, Ube2n/2v1, weakly with Ube2e1, but not with Ube2w (Fig 4B). To assess ubiquitination of the EGFR, the reactions were denatured to separate the Cbl and EGFR proteins (as described in the methods) and the EGFR was immunoprecipitated using an anti-GST antibody (Fig 4C). Ubiquitination of the EGFR can be seen when the EGFR was co-incubated with Cbl and Ube2d2 and Ube2n/2v1, but not with Ube2e1 or Ube2w. Immunoprecipitation of the EGFR but not Cbl under these conditions was confirmed by immunoblotting (Fig 4C, middle and lower panels, respectively). Ube2e1 gave a weak ubiquitination signal in the reactions shown in Fig 4B. To further test whether the Cbl/Ube2e1 pair could mediate ubiquitination of the EGFR, the reactions were repeated using an increased amount of Ube2e1 (500 ng in Fig 4D compared to 250 ng in Fig 4B). The Cbl/Ube2d2 was used as a positive control. Again, Ube2e1 resulted in a weaker ubiquitination signal compared to Ube2d2 (Fig 4D, compare lane 4 to lane 2). The reactions were denatured as in Fig 4C and tested for EGFR ubiquitination (Fig 4E). Weak ubiquitination of the EGFR could be seen when an increased amount of the Ube2e1 was used (Fig 4E, lane 4). Again, the Cbl/Ube2d2 pair was able to ubiquitinate the EGFR (Fig 4E, lane 2). Immunoprecipitation of the EGFR but not Cbl under these conditions was again confirmed by immunoblotting (Fig 4E, middle and lower panels, respectively).

Cbl proteins have been shown to ubiquitinate the non-receptor tyrosine kinase Syk [30–32]. To test Cbl-mediated ubiquitination of Syk, we preincubated recombinant GST-Cbl N1/2 with recombinant full length Syk in the E3 assay buffer without E2 to allow Cbl phosphorylation (Fig 4F). Incubation of the GST-Cbl protein with Syk resulted in phosphotyrosine bands that comigrated with Syk and Cbl (Fig 4F, top panel, lanes 2 and 4). The Syk and GST-Cbl proteins migrate at the same size on the gel such that the phosphotyrosine band seen could be either protein. To confirm that the Cbl protein was phosphorylated, an aliquot of the phosphorylation reaction was denatured to separate the Cbl and Syk proteins and then immunoblotted for phosphotyrosine (Fig 4F, 5^th^ panel). Small amounts of Syk were non-specifically immunoprecipitated by the anti-Cbl antibody (Fig 4F, 7^th^ panel, lanes 2 and 4) but a phosphotyrosine band is only seen when Cbl is precipitated from the samples where Cbl and Syk are coincubated, consistent with Cbl phosphorylation (Fig 4F, 5^th^ panel, lane 4). Aliquots of the Cbl and Syk proteins from the phosphorylation step were used in E3 assays as shown in Fig 4G. In the absence of phosphorylation, no Cbl-mediated ubiquitination was observed (Fig 4G, lane 4). When phosphorylated Cbl was used, ubiquitination was seen with Ube2d2, Ube2n/2v1, weakly with Ube2e1, but not with Ube2w (Fig 4G). To assess ubiquitination of the Syk, an aliquot of each reaction was denatured to separate the Cbl and Syk proteins (as described in the methods) and the Syk was immunoprecipitated using an anti-Syk antibody (Fig 4H). Ubiquitination of Syk can be seen when Syk was co-incubated with Cbl and Ube2d2, Ube2n/2v1, and Ube2e1, but not with Ube2w. Immunoprecipitation of Syk but not Cbl under these conditions was confirmed by immunoblotting (Fig 4H, middle and lower panels, respectively).

Thus, Cbl mediates ubiquitination of the EGFR and Syk *in vitro* when paired with Ube2d2, Ube2n/2v1, and Ube2e1.

### Ubiquitin linkages formed by Cbl are dependent on the E2

**Fig 5:**
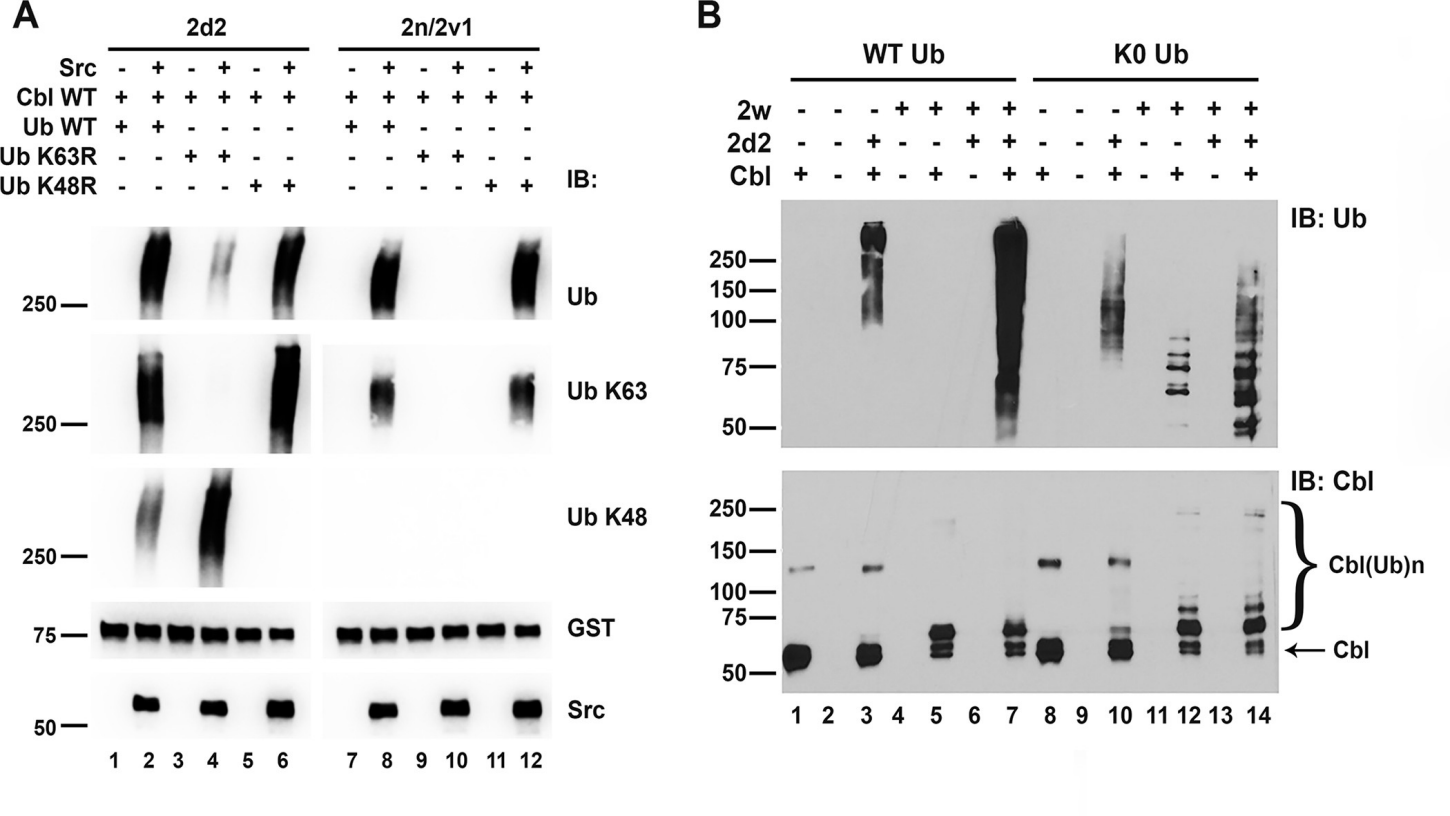
E2 determines the type of ubiquitin links formed by Cbl. **(**A) Ube2d2 or Ube2n/2v1 were incubated with WT GST-Cbl with either WT ubiquitin (Ub), K63R or K48R mutant Ub with or without Src as indicated. Reactions were immunoblotted and probed with anti-Ub, Ub K63-specific, Ub K48-specific, anti-GST and anti-Src antibodies. MW in kDa is shown to the left of the panels. (B) Cbl, cleaved from the GST protein, and Ube2w were incubated for 20 minutes with wild-type ubiquitin (WT Ub) or ubiquitin mutant (K0 Ub) and then Ube2d2 was added to the reaction as indicated. Assays were performed for 40 minutes in total and immunoblotted (IB) for ubiquitin (Ub) and Cbl.

We next tested the types of ubiquitin chains formed by Cbl in the presence of different E2s. Ube2n/2v1 has been reported to support the formation of K63-linked ubiquitin chains [28]. We tested the types of ubiquitin links formed on Cbl in the presence of Ube2n/2v1 as compared to Ube2d2. For that, ubiquitin molecules with either lysine 63 or 48 mutated to arginine were used in the *in vitro* E3 assays. In addition, we used Ub K63- and K48-specific antibodies to detect the ubiquitin chains formed on Cbl. As described above in Fig 3, strong activity of WT GST-Cbl was observed only when Src was added to the reaction (Fig 5A, compare lanes with or without Src). We found that in the presence of Ube2d2, Cbl promoted the formation of both K63- and K48-linked ubiquitin chains, since the replacement of WT Ub with either K63R or K48R still produced the characteristic smear detected by anti-Ub antibodies (Fig 5A, lanes 2, 4 and 6). K63-specific antibodies only detected the ubiquitin smear from the reactions containing WT and K48R ubiquitin, while K48-specific antibodies detected the signal only in the reaction containing WT and K63R ubiquitin, confirming the nature of the ubiquitin chains formed. The Cbl/Ube2d2 pair appears to form K63 linked chains to a greater extent than K48 linked chains (Fig 5A, top panel, compare the reduction in the ubiquitination smear measured by the pan-ubiquitin antibody when K63R vs K48R ubiquitin is used). When the Ube2n/2v1 pair was tested in the same system, ubiquitin chain formation was only observed in the presence of WT and K48R ubiquitin, but not with K63R ubiquitin (Fig 5A, compare lanes 8 and 12 to lane 10). The ubiquitin chains formed in the presence of the Ube2n/2v1 pair were detected with anti-Ub and Ub K63-specific antibodies but not with Ub-K48 specific antibody (Fig 5A), confirming the finding that Ube2n/2v1 guided Cbl to produce K63-linked polyubiquitin chains.

Our yeast two-hybrid screen established that Ube2w can interact with WT and activated Y371E Cbl. However, the E3 assay screens did not identify Ube2w as a specific E2 for Cbl (S1 Fig, Tables 1 and 3). Prior work has suggested that Ube2w can monoubiquitinate proteins and prime them for polyubiquitination by other E2s [24]. Thus, we wanted to test whether Ube2w could modulate Cbl autoubiquitination *in vitro* in the presence of Ube2d2. We employed *two-step in vitro* E3 assays where the Cbl, cleaved from the GST protein, was first incubated with or without Ube2w, and then Ube2d2 was added to the reactions. As shown above in Fig 1, Cbl E3 autoubiquitination activity was stimulated in the presence of Ube2d2 when wild-type ubiquitin was used (Fig 5B, lane 3). Again, we did not see any E3 activity when Cbl was incubated with Ube2w and wild-type ubiquitin (Fig 5B, lane 5). However, there was enhanced E3 activity in the presence of both Ube2w and Ube2d2 compared to Ube2d2 alone (Fig 5B, compare lane 3 to lane 7). Published data has shown that Ube2w can monoubiquitinate its substrates *in vitro* and in cells [24, 33–36]. On the Cbl immunoblot for the reactions with wild-type ubiquitin there appears to be a shift in the size of the Cbl protein consistent with monoubiquitination, but no evidence for this was seen in the ubiquitin blot (Fig 5B, lane 5, lower panel vs upper panel). To more directly test whether Ube2w can monoubiquitinate Cbl in the *in vitro* E3 assay, a ubiquitin with all lysines mutated to arginines (K0) was used (Fig 5B, lanes 8–14). In these assays mono- or multi mono-ubiquitinated Cbl proteins were detected on both the ubiquitin blot and the Cbl blot with Ube2d2 and Ube2w used individually as well as in combination (Fig 5B, lanes 10, 12 and 14, respectively). From these experiments, it appears that Ube2d2 results in higher weight ubiquitinated products, consistent with more sites of mono-ubiquitination, compared to Ube2w (Fig 5B, compare lane 10 to lane 12).

The discordance between the detection of monoubiquitinated Cbl on the Cbl blots for both WT and K0 ubiquitin but on the ubiquitin blots only with K0 ubiquitin suggested that the antibody used (P4D1 from Santa Cruz) detected K0 monoubiquitin with a higher sensitivity than WT monoubiquitin. To test this, we immunoblotted wild-type and K0 monoubiquitin with the P4D1 antibody used above and the DAKO Z0458 antibody (S5 Fig). These immunoblots confirmed that both antibodies detect the K0 monoubiquitin with a higher sensitivity. Since our original E3 assays described in Fig 1, S1 Fig, and S4 Fig were all done with wild-type ubiquitin this suggests that we did not see monoubiquitination by Ube2w in those assays in part due to the antibody issue. This suggests we may have missed some E2s in the initial screen that mediate monoubiqutination with Cbl but since no other E2s were identified in the yeast two-hybrid screen we did not pursue this further. It also suggests that other investigators may miss monoubiquitination using the antibodies shown in S5 Fig.

These data show that *in vitro*, Ube2d2 can initiate and elongate ubiquitin chains formed on Cbl, while Ube2w can only carry out the initiation step. The combination of Ube2d2 and Ube2w results in increased ubiquitination.

Thus, we concluded that the type of ubiquitin links produced by Cbl depends on the E2 present in the reaction: Ube2d2 supports K48- and K63-linked chains, Ube2n/2v1 mediates K63-polyubiqutination, while Ube2w supports only monoubiquitination.

### Knockdown of Ube2d family, Ube2e family, and Ube2n in HeLa cells reduces EGFR ubiquitination

**Fig 6:**
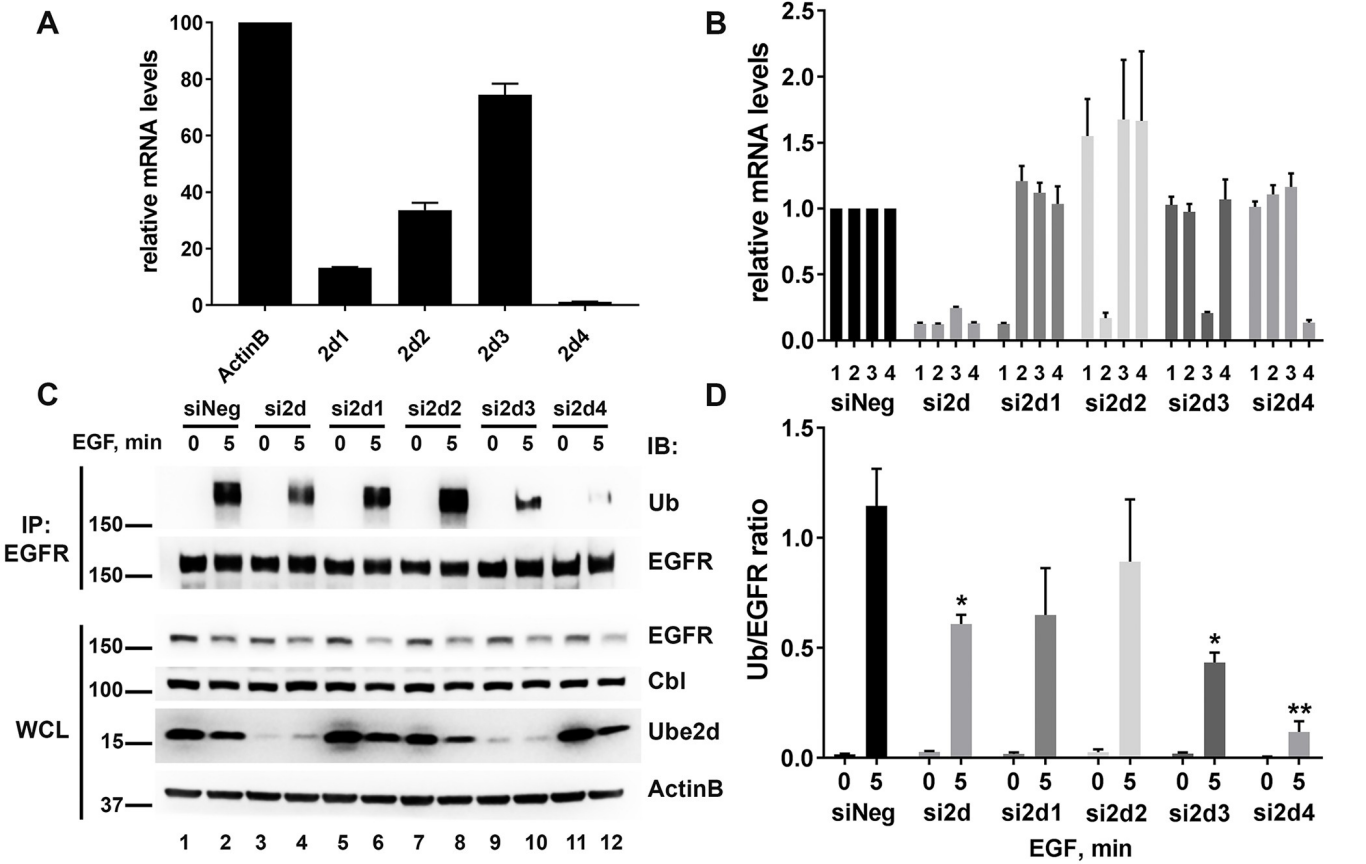
Knockdown of Ube2d proteins decreases ubiquitination of EGFR in HeLa cells. HeLa cells were transfected with control siRNA (siNeg), or siRNAs targeting Ube2d1, Ube2d2, Ube2d3 and Ube2d4 separately or together (si2d) for 48 hours and then were either left untreated (0) or treated with 25 ng/mL EGF for 5 minutes (5). (A) RT-QPCR analysis of Ube2d mRNA expression as compared to ActinB expression in HeLa cells transfected with siNeg averaged for four independent transfections ± SEM. (B) RT-QPCR analysis of Ube2d knockdown efficiency in transfected HeLa cells. Numbers 1 through 4 on the X axis indicate the primers to Ube2d1 through Ube2d4, respectively, used in the PCR reaction. Average for three independent experiments ± SEM is shown. (C) Immunoblot analysis of whole cell lysates (WCL) using antibodies to detect EGFR, Cbl, all members of Ube2d family, and ActinB. EGFR was immunoprecipitated from the lysates and blotted for ubiquitin (Ub) or EGFR as indicated. (D) The fraction of ubiquitinated EGFR was calculated by densitometry analysis of EGFR IP from three independent experiments and plotted as an average ratio of Ub/EGFR ± SEM. Asterisk (*) denotes p < 0.05, (**) denotes p < 0.01. MW in kDa is shown to the left of the WB panels.

We and others have demonstrated that EGFR becomes ubiquitinated upon stimulation of HeLa cells with EGF and the Cbl proteins play a major role in this process, since the knockdown of both Cbl and Cbl-b leads to dramatic reduction in EGFR ubiquitination and prevents EGFR down regulation (S6 Fig). Ube2d family members promoted autoubiquitination of Cbl (Fig 1) as well as Cbl-mediated ubiquitination of EGFR and Syk *in vitro* (Fig 4). Ube2d E2s have previously been shown to function with Cbl in cells to ubiquitinate the activated EGFR in cells [23]. We tested which Ube2d protein functions with Cbl to induce EGFR ubiquitination in HeLa cells. RT-QPCR analysis showed that among Ube2d family, the mRNA expression level of Ube2d3 was the highest in HeLa cells, followed by Ube2d2 and Ube2d1 (Fig 6A). The expression level of Ube2d4 was about 1% of that of ActinB (Fig 6A). We then depleted Ube2d family members together or individually with specific siRNAs. The efficient knockdown of each protein was confirmed by RT-QPCR as there are no antibodies that selectively recognize the individual family members (Fig 6B). Importantly, each siRNA used was specific and did not affect the mRNA expression of other Ube2d proteins. Analysis of HeLa whole cell lysate (WCL) confirmed the finding that Ube2d3 is the most abundant form in Hela cells, since its depletion led to substantial decrease in signal detected by an anti-Ube2d family antibody that recognizes all 4 human Ube2d proteins (Fig 6C). siRNA transfected cells were either starved (0 min EGF) or treated with EGF for 5 min to induce EGFR ubiquitination. EGFR was then immunoprecipitated from the lysates and subjected to IB with anti-ubiquitin and anti-EGFR antibodies. We observed robust EGFR ubiquitination in the EGF stimulated control (siNeg) cells and this was decreased by 47% with the depletion of all Ube2d proteins (Fig 6C and 6D). Despite the effects on ubiquitination seen with knockdown of the Ube2d protein family, there was no significant change in the degree of EGFR down regulation as measured by protein levels (Fig 6C). When the contribution of each Ube2d protein was analyzed individually, knockdown of Ube2d3 and Ube2d4 decreased EGF-induced EGFR ubiquitination by 62% and 90%, respectively, while Ube2d2 had no significant effect (Fig 6D). Knockdown of Ube2d1 also decreased EGFR ubiquitination, but this effect was not statistically significant (p = 0.14). Thus, we confirmed the previously published finding that Ube2d family members act as E2s for Cbl in cells [23]. Moreover, for the first time, we demonstrated that Ube2d3 and Ube2d4 are the main contributors to the EGFR ubiquitination in HeLa cells. Surprisingly, Ube2d4, being the least abundant protein in HeLa cells, judged by its mRNA expression and the effect of the siRNA on the total Ube2d protein level, contributed the most to EGFR ubiquitination.

**Fig 7:**
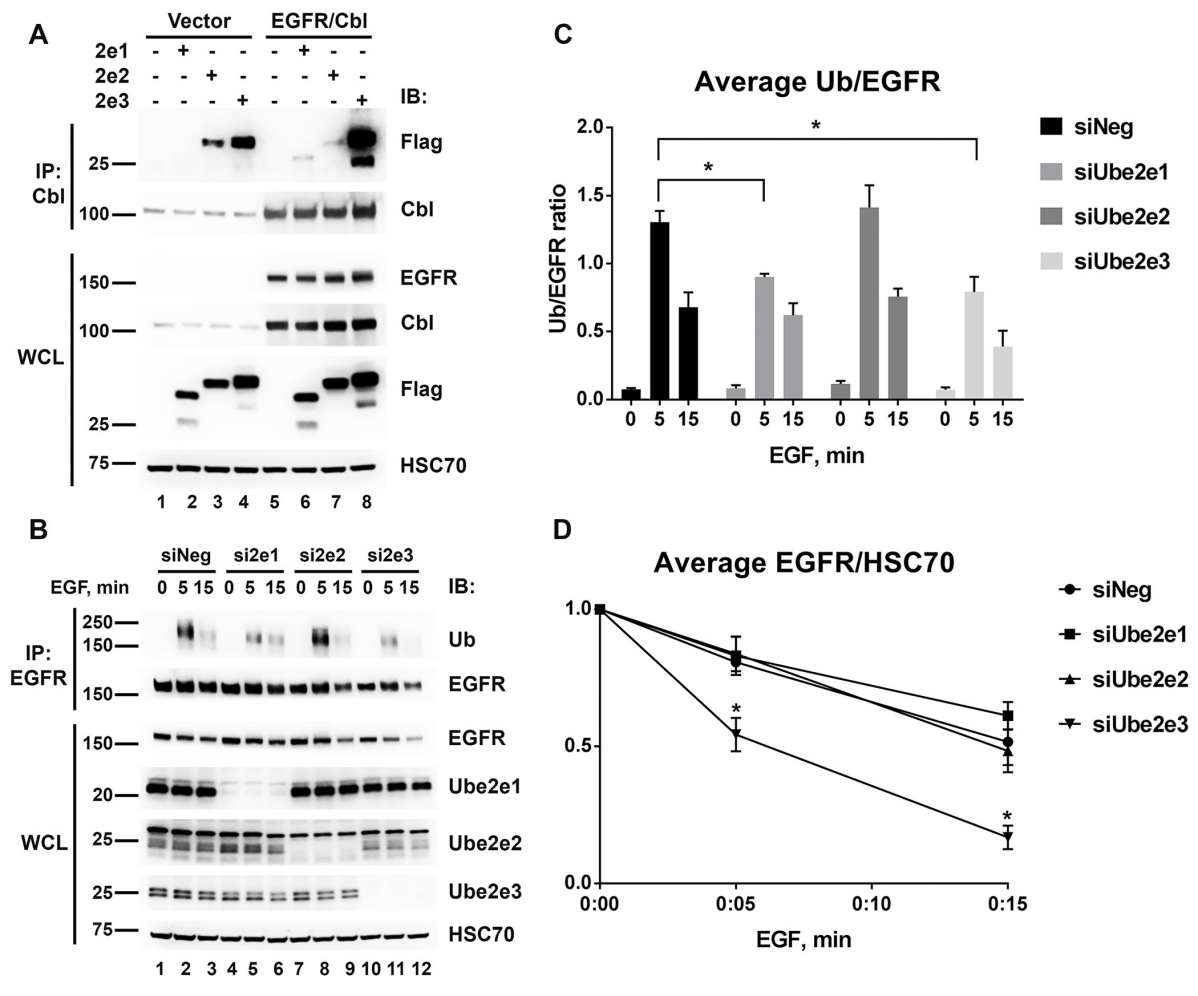
Ube2e1 and Ube2e3 interact with Cbl and modulate EGFR ubiquitination in cells. (A) HEK293T cells were transfected with human Flag-tagged Ube2e1, Ube2e2, Ube2e3 in combination with empty vector or EGFR/Cbl as indicated. Cbl was immunoprecipitated with anti-Cbl antibodies to detect the interaction with Ube2e family proteins (Flag). Whole cell lysate (WCL) was immunoblotted to detect EGFR, Cbl and Flag-tagged Ube2e proteins. HSC70 was used as a loading control. (B) HeLa cells were transfected with control siRNA (siNeg) or with siRNA targeting Ube2e1, Ube2e2, Ube2e3 for 48 hours and then treated with 25 ng/ml of EGF for the indicated time periods. EGFR was immunoprecipitated and probed with anti-ubiquitin (Ub) and anti-EGFR antibodies. Knockdown of individual Ube2e proteins was confirmed by probing whole cell lysates (WCL) with the specific antibodies as indicated to the right of the panels. (C) The fraction of ubiquitinated EGFR for each treatment was calculated by densitometry analysis of EGFR IP from five independent experiments and plotted as an average ratio of Ub/EGFR ± SEM. (D) Degradation of EGFR upon EGF stimulation was calculated by densitometry analysis of WCL from five independent experiments and plotted as an average ratio of EGFR/HSC70 ± SEM. EGFR levels in untreated samples (0 EGF) were set as 1. Asterisk (*) denotes p < 0.01 as compared to siNeg. MW in kDa is shown to the left of the panels.

Ube2e proteins supported the autoubiquitination of Cbl and Cbl-mediated ubiquitination of EGFR and Syk *in vitro*, thus suggesting that they might serve as cognate E2s for Cbl and possibly contribute to the ubiquitination of Cbl substrates in cells. To test the interaction between Ube2e family members and Cbl in human cells, HEK293T cells were transfected with EGFR, Cbl, empty vector (control) and Flag-tagged Ube2e1, Ube2e2 or Ube2e3 (Fig 7A). The overexpression of all the plasmids was confirmed by probing HEK293T whole cell lysates (WCL) with anti-Flag antibody. Cbl was immunoprecipitated and probed with anti-Flag antibody to detect individual Ube2e proteins. Immunoprecipitation of Cbl with anti-Cbl antibody brought down both endogenous and overexpressed Cbl (Fig 7A, lanes 1–4 and 5–8, respectively). As expected, overexpressed Cbl was detected in the immune precipitate more readily than the endogenous protein (compare lanes 5–8 to lanes 1–4). All three Ube2e members were co-precipitated with Cbl, however only the amount of Ube2e1 and Ube2e3 proteins in the Cbl IP increased in cells overexpressing Cbl, indicating the specific interaction between the proteins (compare lane 6 to lane 2 and lane 8 to lane 4, respectively). Ube2e2 did not increase when Cbl was immunoprecipitated from cells overexpressing Cbl, suggesting that Ube2e2 was immunoprecipitated by anti-Cbl antibodies non-specifically (compare lane 7 to lane 3). The interactions between individual Ube2es and Cbl were very weak, as it took maximal signal amplification to detect Ube2es in Cbl IP. Thus, we concluded that Ube2e1 and Ube2e3, but not Ube2e2 interact with Cbl in cells.

We next tested whether the Ube2e family of E2s could modulate Cbl-mediated EGFR ubiquitination and degradation. HeLa cells were transfected with either siRNAs targeting the individual members of Ube2e family or control nontargeting siRNA (siNeg) for 48 hours, and then treated with EGF. Knockdown efficiency of Ube2e siRNAs was confirmed by western blot with specific antibodies (Fig 7B, WCL). To determine whether EGFR ubiquitination was affected by the knockdown of individual Ube2es, EGFR was immunoprecipitated from unstimulated and EGF stimulated HeLa cells and probed for ubiquitin. The data was quantified and plotted as normalized intensities of Ub bands to EGFR bands of EGFR IP (Fig 7C). EGF-induced EGFR ubiquitination peaked at 5 min, and then decreased by 15 min of stimulation. Knockdown of Ube2e1 and Ube2e3 decreased EGFR ubiquitination after 5 min of EGF stimulation by 31% and 40%, respectively. Knockdown of Ube2e2 had no effect on EGFR ubiquitination. To quantify EGF-induced degradation of EGFR, the intensities of EGFR in WCL were normalized to the corresponding intensities of loading control (HSC70) and plotted versus EGF stimulation time (Fig 7D). EGFR levels in untreated samples (0 EGF) were set as 1. Interestingly, knockdown of individual Ube2es had differential effect on EGFR degradation (Fig 7B and 7D). Knockdown of Ube2e1 and Ube2e2 did not affect EGFR degradation, but the knockdown of Ube2e3, which results in less ubiquitination, led to increased EGFR degradation compared to control cells. This increased degradation might be due to the off-target effects of Ube2e3 siRNA. Other possibilities are addressed in the discussion section below. Thus, the data indicate that Ube2e1 and Ube2e3 act as specific E2s for Cbl in cells and contribute to the ubiquitination of EGFR.

**Fig 8:**
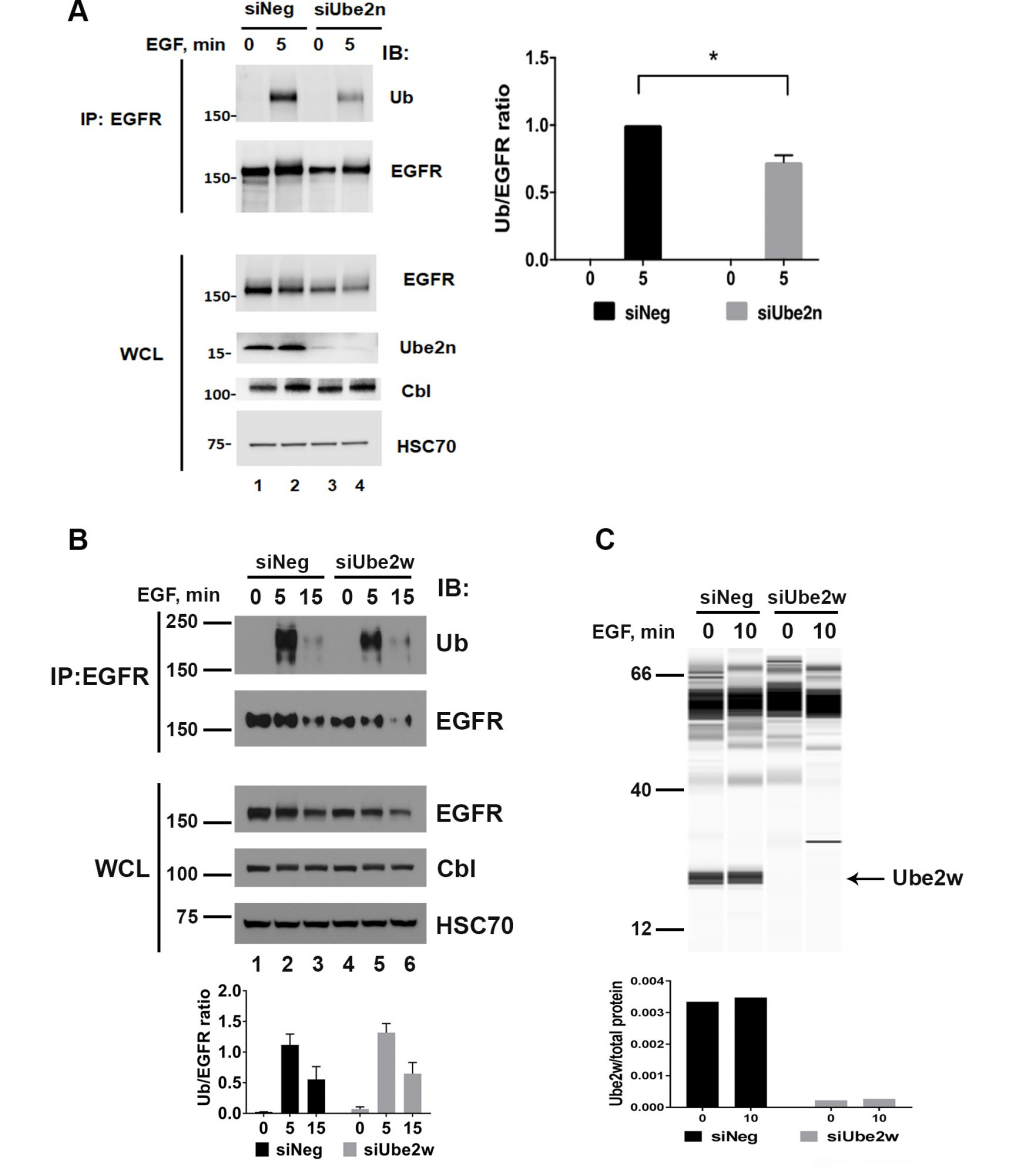
Ube2N but not Ube2w modulates Cbl-mediated ubiquitination of the EGFR in cells. (A) Knockdown of Ube2n in HeLa cells reduces EGFR ubiquitination. HeLa cells were transfected with control siRNA (siNeg) or siRNA targeting Ube22n for 72 hours and then treated with EGF (25 ng/mL) for 5 min. Whole cell lysates (WCL) were subjected to western blot analysis using antibodies to detect Ube2n, EGFR, Cbl and HSC70 as indicated. EGFR was immunoprecipitated from the lysates and blotted for ubiquitin (Ub) or EGFR as indicated. The graph under the WB panels shows an average ratio of Ub/EGFR ± SEM calculated by densitometry analysis of EGFR IP and normalized to the siNeg for each experiment for three independent experiments. EGFR ubiquitination in siUbe2n transfected cells was statistically lower (*p<0.05) than in siNeg transfected cells after 5 min of EGF stimulation. (B) Knockdown of Ube2w in HeLa cells did not affect EGFR ubiquitination. HeLa cells were transfected with control siRNA (siNeg) or siRNA targeting Ube2w for 48 hours and then treated with EGF (25 ng/mL) for the indicated time periods. Whole cell lysates (WCL) were subjected to western blot analysis using antibodies to detect EGFR, Cbl and HSC70 as indicated. EGFR was immunoprecipitated from the lysates and blotted for ubiquitin (Ub) or EGFR as indicated. The graph under the WB panels shows an average ratio of Ub/EGFR ± SEM calculated by densitometry analysis of EGFR IP for four independent experiments. EGFR ubiquitination in siUbe2w transfected cells was not statistically different from siNeg transfected cells after 5 or 15 min of EGF stimulation. (C) Ube2w knockdown was confirmed by detection of the protein using Simple Western capillary electrophoresis assay. The upper panel shows Ube2w protein levels on a simulated immunoblot of HeLa cells transfected as in B and stimulated with EGF as indicated, the graph below shows the quantification of Ube2w levels with and without EGF stimulation (0 and 10 min) normalized to the total protein levels. MW in kDa is shown to the left of the panels.

The Ube2n/2v1 E2 pair also supported ubiquitination of the EGFR *in vitro* (Fig 4C). Ube2n was knocked down in HeLa cells and the effects on EGFR ubiquitination assessed (Fig 8A). When Ube2n was silenced, there was a statistically significant 30–40% reduction of the ubiquitination of the EGFR (Fig 8A, compare lane 4 to lane 2 and quantified below the blots). Thus, Ube2n can contribute to ubiquitination of EGFR in cells. There was no effect on EGFR degradation with the loss of Ube2n.

Finally, to test whether Ube2w affects Cbl E3 activity in cells, we investigated the effects of Ube2w knockdown on EGFR degradation and ubiquitination. HeLa cells were transfected with a Ube2w siRNA for 48 hours and then treated with EGF for the indicated times (Fig 8B). There was no obvious change in EGFR degradation upon EGF treatment when Ube2w was depleted (Fig 8B, compare lanes 4–6 to lanes 1–3). Ubiquitination of the EGFR was assessed by immunoprecipitation of the EGFR and immunoblotting with anti-ubiquitin antibodies. There was no statistically significant change in EGFR ubiquitination in Ube2w knockdown cells in comparison with corresponding control cells (Fig 8B, compare lanes 5–6 and 2–3, the quantification is shown below). Immunoblotting using a Simple Western automated capillary electrophoresis assay demonstrated that Ube2w knockdown efficiency was at least 90% (Fig 8C). Thus, our data indicated that Ube2w did not contribute to Cbl-mediated EGFR ubiquitination in cells.

## Discussion

Understanding the function of an E3 depends in part on knowledge of the E2s with which it interacts [24, 37–40]. We sought to characterize E2 proteins that interact with Cbl ubiquitin ligase, since this E3 plays an important role in the regulation of RTK signaling and ultimately cell proliferation [1, 2]. In published data, although Ube2d1, Ube2d2, Ube2d3 and Ube2l3 were found to interact with Cbl, only Ube2d family members could support Cbl E3 activity *in vitro* and in cells [19–23]. In this study, we employed an *in vitro* autoubiquitination assay and a yeast two-hybrid screen to identify the full spectrum of E2s that cooperate with Cbl *in vitro* to mediate both autoubiquitination and substrate ubiquitination. We then verified that some of the identified E2s do indeed contribute to the ubiquitination of the Cbl substrate, EGFR, in cells.

In our initial *in vitro* screen (Fig 1) we used mutant recombinant Cbl Y371E, since this phospho-mimetic Y to E mutation in the linker tyrosine has been shown to increase the activity of the Cbl proteins when tested in *in vitro* autoubiquitination assays [11, 12]. Even with this activated Cbl in the reaction only Ube2d family members could mediate autoubiquitination of Cbl (Fig 1). However, when the protocol was modified to include WT Cbl and tyrosine kinase Src into the reaction, three members of Ube2e family as well as Ube2n/2v1 were found to be active in Cbl autoubiquitination, although this activity was weaker compared to Ube2d proteins (Fig 4 and Table 3). Previous work by us and others has shown that Src phosphorylates Cbl proteins on the conserved linker tyrosine (at Y371 for Cbl) and thus enhances its E3 activity [11, 12, 22, 28]. Our failure to identify the activity of the Ube2e E2s and Ube2n/2v1 in the initial screen was due to greater enhancement of Cbl E3 activity by phosphorylation of the linker tyrosine compared to that seen with the phospho-mimetic Y to E mutation in the linker tyrosine. Indeed, more ubiquitination was observed with Src phosphorylated WT Cbl when its activity was directly compared to that of Y371E mutant (Fig 3 and S4 Fig). Supporting our finding, recent studies identified that Ube2d2-Ub Kd for the Cbl Y371E mutant was higher than for phosphorylated Cbl, measuring 9 and 2.6 μM, respectively [41]. In addition, Src itself may serve as a substrate for Cbl, thus further accounting for the increase in the overall ubiquitination observed with the addition of Src to the E3 assay reaction [42].

Surprisingly, neither the Ube2d E2s (*a*.*k*.*a*., UbcH5a-c) nor Ube2l3 (*a*.*k*.*a*., UbcH7) interacted with Cbl in the yeast two-hybrid assay (Figs 2, S2 Fig, and S3 Fig). A recent yeast two-hybrid screen of human E2/E3-RING interactions failed to identify interactions between WT Cbl and any of the 39 E2 and E2-like proteins [43]. Ube2l3 has been co-crystallized with the Cbl RF [19], but did not support autoubiquitination of Cbl *in vitro* [21]. Ube2l3’s inability to ubiquitinate Cbl is consistent with its being a dedicated E2 for HECT type and RING-in between-RING type E3s, as Ube2l3 can only transfer its ubiquitin to cysteine residue, not to lysine, as would be required for a RING E3, such as Cbl [44]. Ube2d E2s support autoubiquitination by Cbl in the *in vitro* assay (Fig 1) and Ube2d2 was co-crystalized with Cbl [21, 28]. Published data suggested that common methods, such as co-immunoprecipitation, may not be effective for identification of protein-protein interactions for evaluating all E2-E3 pairs since E2-E3 interactions are transient and of low affinity [21, 24, 45, 46]. Consistent with this, biophysical analysis of the complex of Ube2d2 with Cbl found the Kd for WT Cbl and phosphorylated Cbl to be 311 and 28 μM, respectively [28]. Thus, the interaction between these E2s and Cbl proteins may be too weak to be detected in yeast interaction assay.

We tested the ability of Cbl to ubiquitinate known substrates EGFR and Syk using recombinant proteins *in vitro* (Fig 4). As we demonstrated with Src (Fig 3A), active EGFR (Fig 4A) and Syk (Fig 4F) could phosphorylate Cbl *in vitro*. Using the EGFR or Syk phosphorylated Cbl proteins, we found that Ube2d2, Ube2n/2v1, and Ube2e1 could all mediate ubiquitination of the EGFR (Fig 4C and 4E) and Syk (Fig 4H). Consistent with the results for autoubiquitination (Fig 3), Ube2e1 resulted in less ubiquitination than Ube2d2. We did not observe any ubiquitination of EGFR or Syk when Ube2w was included in the assays (Fig 4). Together, the data identified multiple E2s that could function with Cbl to mediate autoubiquitination and substrate ubiquitination *in vitro*.

We also found that the different E2s resulted in different ubiquitin linkages when tested with Cbl *in vitro* (Fig 5). Ube2d2 resulted in both K48 and K63 linked ubiquitin chains as determined both by utilization of specific ubiquitin mutants (*i*.*e*., K48R and K63R) and by blotting with linkage specific anti-ubiquitin antibodies (Fig 5A). In our assays, the predominant chains formed with the Cbl/Ube2d2 pair appears to be K63 linked polyubiquitin (Fig 5A). Prior work has shown that the Ube2d E2s can form both K48 and K63 ubiquitin linkages [47, 48]. In contrast the Ube2n/2v1 only formed K63 linked ubiquitin chains when incubated with Cbl and this again is consistent with prior findings [24, 47]. Finally, we found that Ube2w, which interacted with Cbl in the yeast two-hybrid screen, could mediated monoubiquitination when incubated with Cbl (Fig 5B). Thus, the E2 directs the type of ubiquitin chain formed by the Cbl E3. Others have reported that the E2 specifies the type of ubiquitin chains formed by E3 proteins [24, 47].

Ube2w interacted with Cbl in yeast two-hybrid screen in a RF dependent fashion (Fig 2), could monoubiquitinate Cbl, and enhanced autoubiquitination of Cbl in combination with Ube2d2 (Fig 5B). We did not see monoubiquitination by Ube2w in our initial screen (S1 Fig). In the initial screening E3 assays (Fig 1 and S1 Fig) we used a GST-Cbl N-terminal construct, while in the experiments in Fig 5B we used a Cbl construct that was cleaved from the GST fusion protein. It has been demonstrated that Ube2W has a unique ability to modify the α-amino group of the proteins with intrinsically disordered N-termini [49]. Our data suggests that N-terminus of Cbl, but not that of GST, meets the requirement for Ube2w monoubiquitinating activity. Our finding of Ube2w’s ability to transfer monoubiquitin to a substrate is in agreement with the published literature [14, 24, 33, 34, 36]. Ube2W was found to attach monoubiquitin to RING E3 TRIM5α, which can then act as a substrate to anchor K63-linked polyubiquitin chains built by Ube2n/Ube2v2 *in vitro* and in cells [50]. Ube2w also attached 1–3 ubiquitin molecules to U-box E3 ligase CHIP *in vit*ro. Moreover, when Ube2w was used in combination with Ube2n/Ube2v1, CHIP was polyubiquitinated [51]. BRCA1-BARD1 protein monoubiquitinated by Ube2w in *in vitro* E3 assays served as a substrate for Ube2k as well as Ube2n/Ube2v2 to form K48- or K63-linked ubiquitin chains, respectively [24]. In our assay, Cbl monoubiquitinated by Ube2w served as a substrate for Ube2d2 to result in a greater efficiency of ubiquitination than achieved by Ube2d2 alone. The apparent deficiency of Ube2w in building polyubiquitin chains stems from its inability to ubiquitinate the well-ordered N-terminus of ubiquitin [49].

Ubiquitination of EGFR has been extensively studied in the last two decades, yet our understanding of its mechanisms is far from complete [52]. The silencing of Cbl and Cbl-b confirmed the critical role of Cbl proteins in EGFR ubiquitination (S6 Fig). We found that silencing of the Ube2d family (Fig 6), the Ube2e family (Fig 7), and Ube2n/2v1 (Fig 8A), resulted in decreased ubiquitination of the EGF stimulated EGFR in HeLa cells. EGFR ubiquitination was unaffected by the knockdown of Ube2w in HeLa cells (Fig 8B and 8C) possibly because the N-terminus of EGFR is extracellular and thus unavailable for Ube2w-dependent ubiquitination. Ube2w/Cbl complex may nonetheless be implicated in the monoubiquitination of CIN85 or even Cbl itself. It is also possible that the important role Ube2w plays in many physiological processes such as protein equilibrium, epidermis formation and function of the immune system may stem in part from its interaction with Cbl [53].

We did not see decreased EGFR degradation with the loss of the different E2s despite a decrease in ubiquitination (Figs 6–8). Work by Haglund *et al*. and Mosesson *et al*. demonstrated that even a single monoubiquitin is sufficient to mediate the down regulation of the EGFR [54, 55]. The redundancy of E2s that cooperate with Cbl to mediate EGFR ubiquitination would account for why the loss of any individual E2 or family of E2s did not results in a defect in EGFR down regulation. Down regulation of activated receptors by monoubiquitination was first described in yeast indicating that this is an evolutionarily conserved regulatory mechanism [56–58].

In a landmark study, EGFR was found to be modified by both mono and polyubiquitin at 1:1 ratio with K63-linked ubiquitin being the predominant linkage type [59]. Our results in HeLa cells indicate that Cbl is the main E3 for EGFR responsible for all ubiquitin modifications (S6 Fig). Consistently, Cbl was found to promote both mono and polyubiquitination *in vitro* and mediate formation of both K48 and K63 ubiquitin chains (Fig 5). In cells, Cbl ligates monoubiquitin to CIN85 and EGFR [54, 60] and polyubiquitin to other substrates, including EGFR [23, 61]. Our data show that Cbl forms K63 linked ubiquitin chains with either Ube2d2 and Ube2n/Ube2v1 consistent with the observed results reported by Huang et al. [59]. Thus, our work along with that of others [23], has established that multiple E2s (Ube2d, Ube2e, and Ube2n/2v1) contribute to ubiquitination of the activated EGFR in cells.

Published work has shown that silencing the Ube2d E2 family decreased Cbl-mediated ubiquitination of the activated EGFR in HeLa cells [23] We showed that simultaneously silencing the Ube2d family (Ube2d1, 2d2, 2d3 and 2d4) resulted in a significant decrease in EGFR ubiquitination, confirming the published work (Fig 6). Our further analysis of the Ube2d family in cells found that Ube2d3 and Ube2d4 silencing has the most significant impact on Cbl-mediated ubiquitination of the EGFR (Fig 6). Ube2d3 is the most abundant of the Ube2ds based both on mRNA (Fig 6A and 6B) and on the effects of silencing on the protein levels detected by the pan Ube2d antibody (Fig 6C). Interestingly, the loss of Ube2d4 had the largest impact despite relatively low mRNA and protein levels (Fig 6A, 6B and 6C). Silencing of Ube2d1 and Ube2d2 reduced ubiquitination but the results were not statistically significant. The basis for varying effects of the different Ube2d family members requires further investigation but could be the result of both protein levels and different affinities of the Ube2d family members for Cbl.

All three Ube2e proteins were identified in yeast two-hybrid screen in association with activated phospho-mimetic mutant Cbl (Y371E) and this interaction was also dependent on an intact RF. However, in cells only Ube2e1 and Ube2e3 interacted with Cbl (Fig 7A). Consistently, depletion of Ube2e1 and Ube2e3 decreased ubiquitination of EGFR (Fig 7B and 7C). We observed that despite the decrease in ubiquitination with knockdown of Ube2e3, EGFR degradation was accelerated (Fig 7D). This may represent an off-target effect or it is possible that there are other substrates of Ube2e3 that modulate Cbl-mediated down regulation of EGFR. This will require further investigation.

Although the prevailing models assume a high degree of specificity in the E2-E3 interaction, it has been reported that multiple E2s may interact with a specific E3 *in vitro*. Extensive analysis of human E2 protein interactome revealed that the majority of RING-E3s interact with members of both Ube2d and Ube2e families [43]. The authors of the study concluded that these two families might be responsible for the high proportion of ubiquitination events in human cells. Consistently, we found that these two families of E2s contributed to the ubiquitination of EGFR (Figs 6 and 7). When both Ube2d2 and any of the Ube2e proteins were used in *in vitro* assay, the overall ubiquitination of Cbl was reduced compared to when only Ube2d2 was used as an E2 in the reaction (S4A Fig). This might suggest the competition between the two E2s either for E1 or for Cbl. However, in HeLa cells the two families of E2s work to ubiquitinate EGFR independently without apparent competition, which might be due to a transient nature of E2/Cbl interaction. Additionally, there might exist a tight spaciotemporal regulation of these E2s through the series of scaffold proteins as well as posttranslational modifications [14]. It has been reported that all three members of Ube2e family are imported into the nucleus through the interaction of Ub-charged E2 and importin-11, thus regulating the cytoplasmic/nuclear pool of the proteins [62]. Ube2e1 was found to be covalently modified by ISG15, a ubiquitin-like molecule induced by interferon treatment, and this ISGylation suppressed the ubiquitin charging of Ube2e1 [63]. Ube2e3’s ability to build polyubiquitin chains might be inhibited by non-covalent binding of ubiquitin to the backside residues of E2 [64]. Moreover, N-terminal extension of Ube2e proteins may directly restrict their ubiquitin-conjugating activity to monoubiquitination [65].

Overall our *in vitro* data show that Cbl proteins can interact and function with multiple E2s. In cells, our data indicate the role of Ube2d family, Ube2e family, and Ube2n/2v1 E2s in the regulation of EGFR ubiquitination.

## Supporting information

S1 Fig *In Vitro* E3 autoubiquitination assays. 28 mammalian E2s were tested in an *in vitro* E3 assay for their ability to mediate autoubiquitination by GST-Cbl Y371E (A), GST-Cbl-b Y363E (B), GST-Cbl-c Y341E (C). For Cbl-b and Cbl-c only representative blots are shown. Reactions containing the indicated E2, GST-Cbl construct or GST protein were incubated for 40 min as described in the materials and methods and then parallel gels were immunoblotted for ubiquitin (Ub, top panel) or GST (bottom panel). E2 proteins are listed along the top of the gels and each was tested with GST-Cbl construct (C) or GST alone (G). MW in kDa is shown to the left of the panels.
(TIF)

S2 Fig Identification of E2s that interact with wild-type Cbl. (A) E2 screening with WT Cbl proteins by yeast two-hybrid assay. Cbl expression plasmids were transformed together with one of the 29 E2 plasmids into yeast. Transformed yeasts were diluted 2 times and 10 times and then plated on either -His, -Leu, -Trp, +3AT interaction selection medium (upper panels) or -Leu -Trp medium to confirm that the yeasts received both plasmids (lower panels). The key is shown above the plates. BC112-F-BD115 and BC112-F-BD115 I26A (I*A) with Ube2ds were used as positive and negative controls, respectively. Selected E2s were tested for their ability to interact with Cbl-b (B) and Cbl-c (C) proteins. Cbl-b and Cbl-c constructs were mutated (C373A and C351A, respectively) to test the RF dependence of the interaction.
(TIF)

S3 Fig Identification of E2s that interact with activated Cbl Y371E. E2 screening with activated Cbl Y371E (MT) protein by yeast two-hybrid assay. Cbl Y371 expression plasmids were transformed together with each of 29 E2s plasmids into yeast. Transformed yeasts were plated on either -His, -Leu, -Trp, +3AT interaction selection medium (upper panels) or -Leu -Trp medium to confirm that the yeasts received both plasmids (lower panels). The key is shown above the plates. BC112-F-BD115 and BC112-F-BD115 I26A (I*A) with Ube2ds were used as positive and negative controls, respectively.
(TIF)

S4 Fig Ube2d family, Ube2e family and Ube2n/2v1 support Cbl-mediated autoubiquitination in the presence of Src. (A) *In vitro* E3 assays were performed with Ube2e1, Ube2e2 and Ube2e3 respectively in absence or presence of Ube2d2 or Src as indicated. (B) *In vitro* E3 assays were performed with Ube2d1, Ube2d2, Ube2d3, or Ube2n/2v1 in absence or presence of Src as indicated. Assays were performed with GST-Cbl for 60 min and then immunoblotted with antibodies against ubiquitin, Src, and GST. MW in kDa is shown to the left of the panels.
(TIF)

S5 Fig Ubiquitin antibodies recognize lysine deficient ubiquitin (K0) better than wildtype ubiquitin (Ub). (A) Equal amounts of wildtype or K0 ubiquitin recombinant proteins were immunoblotted with the anti-ubiquitin antibodies P4D1 from Santa Cruz or Z0458 from Dako as indicated. (B) Equal amounts of wildtype or K0 ubiquitin recombinant proteins were separated by PAGE and then stained with Gelcode Blue Stain (ThermoFisher Scientific) to demonstrate equal loading. The immunoblots in A were direct 1000-fold dilutions of the samples run in B.
(TIF)

S6 Fig Cbl and Cbl-b are the major ubiquitin ligases for EGFR in HeLa cells. Hela cells were transfected with siRNA targeting Cbl, Cbl-b or both proteins for 48 hours. The cells were either left untreated (0) or treated with 100 ng/mL EGF for 10 or 30 minutes. EGFR was immunoprecipitated and analyzed for ubiquitination with anti-Ub antibodies. The efficient knockdown of each protein was demonstrated in whole cell lysates (WCL) by probing with anti-Cbl and anti-Cbl-b antibodies. MW in kDa is shown to the left of the panels.
(TIF)
