# Supplementary figures and images for: Cbl interacts with multiple E2s in vitro and in cells
Source: PLoS One. 2019 May 23;14(5):e0216967. doi: 10.1371/journal.pone.0216967 (PMC6533038; doi:10.1371/journal.pone.0216967)

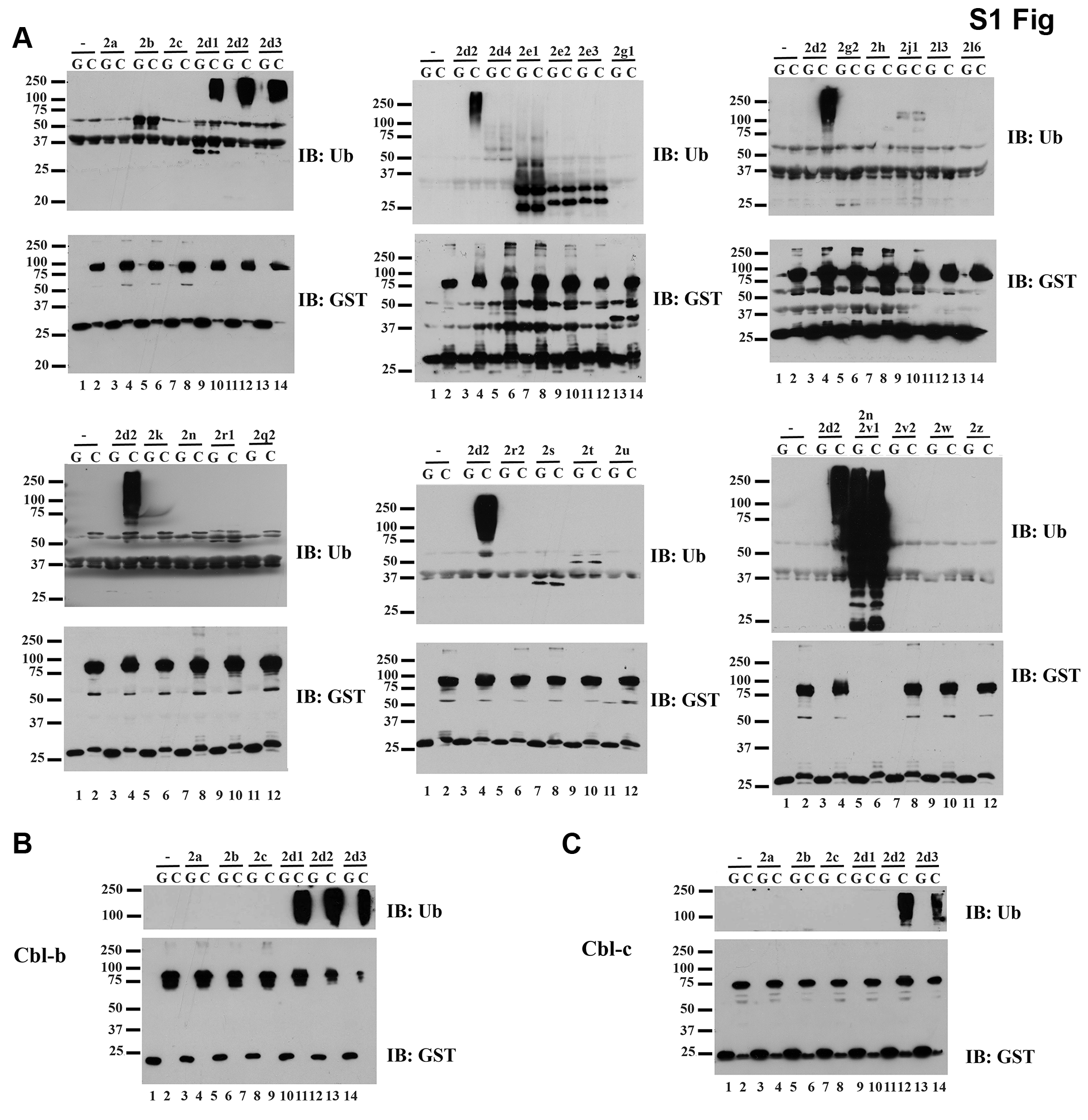

Supplement: S1 Fig — 28 mammalian E2s were tested in an in vitro E3 assay for their ability to mediate autoubiquitination by GST-Cbl Y371E (A), GST-Cbl-b Y363E (B), GST-Cbl-c Y341E (C). For Cbl-b and Cbl-c only representative blots are shown. Reactions containing the indicated E2, GST-Cbl construct or GST protein were incubated for 40 min as described in the materials and methods and then parallel gels were immunoblotted for ubiquitin (Ub, top panel) or GST (bottom panel). E2 proteins are listed along the top of the gels and each was tested with GST-Cbl construct (C) or GST alone (G). MW in kDa is shown to the left of the panels. (TIF) [file pone.0216967.s001.tif]

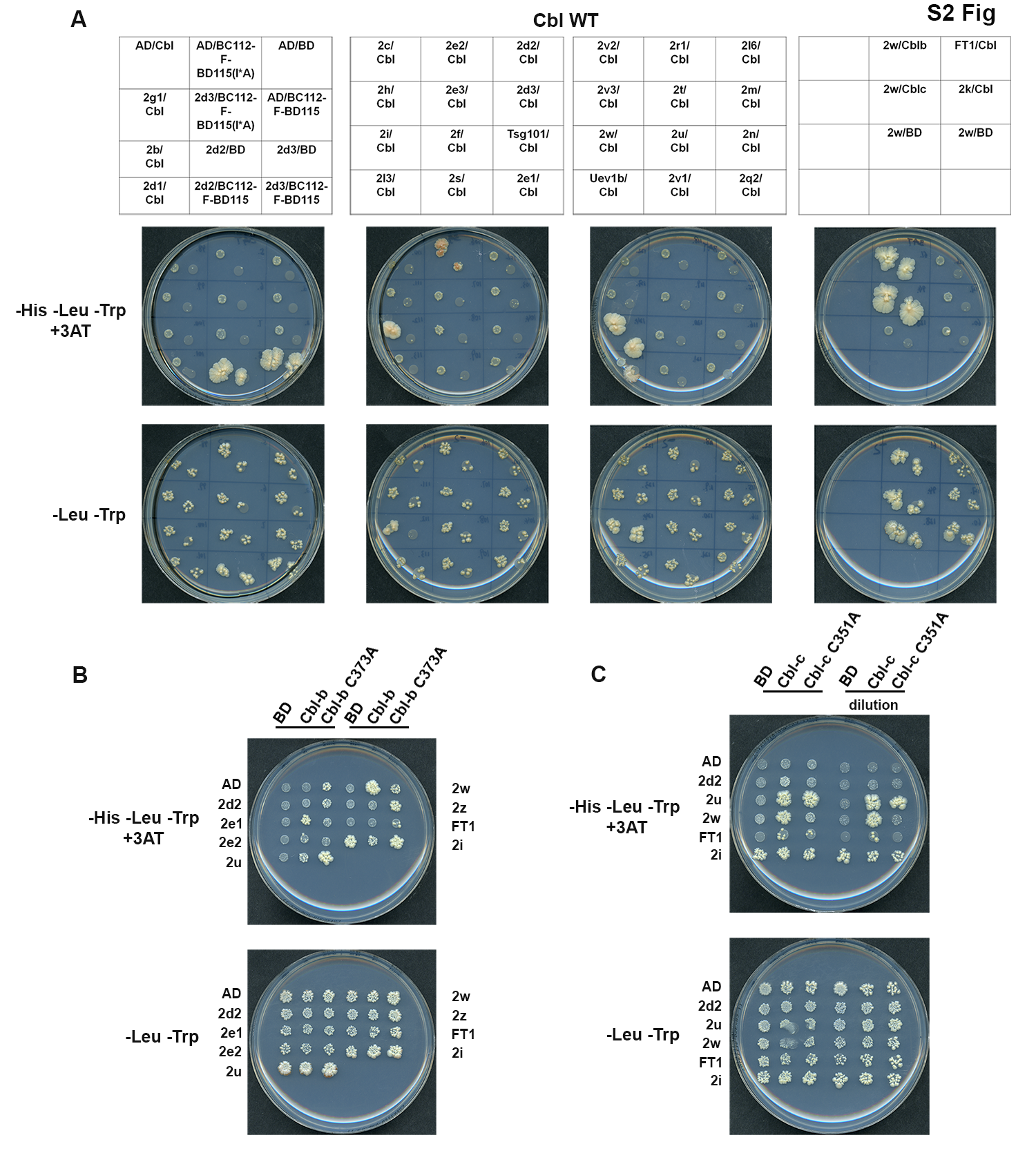

Supplement: S2 Fig — (A) E2 screening with WT Cbl proteins by yeast two-hybrid assay. Cbl expression plasmids were transformed together with one of the 29 E2 plasmids into yeast. Transformed yeasts were diluted 2 times and 10 times and then plated on either -His, -Leu, -Trp, +3AT interaction selection medium (upper panels) or -Leu -Trp medium to confirm that the yeasts received both plasmids (lower panels). The key is shown above the plates. BC112-F-BD115 and BC112-F-BD115 I26A (I*A) with Ube2ds were used as positive and negative controls, respectively. Selected E2s were tested for their ability to interact with Cbl-b (B) and Cbl-c (C) proteins. Cbl-b and Cbl-c constructs were mutated (C373A and C351A, respectively) to test the RF dependence of the interaction. (TIF) [file pone.0216967.s002.tif]

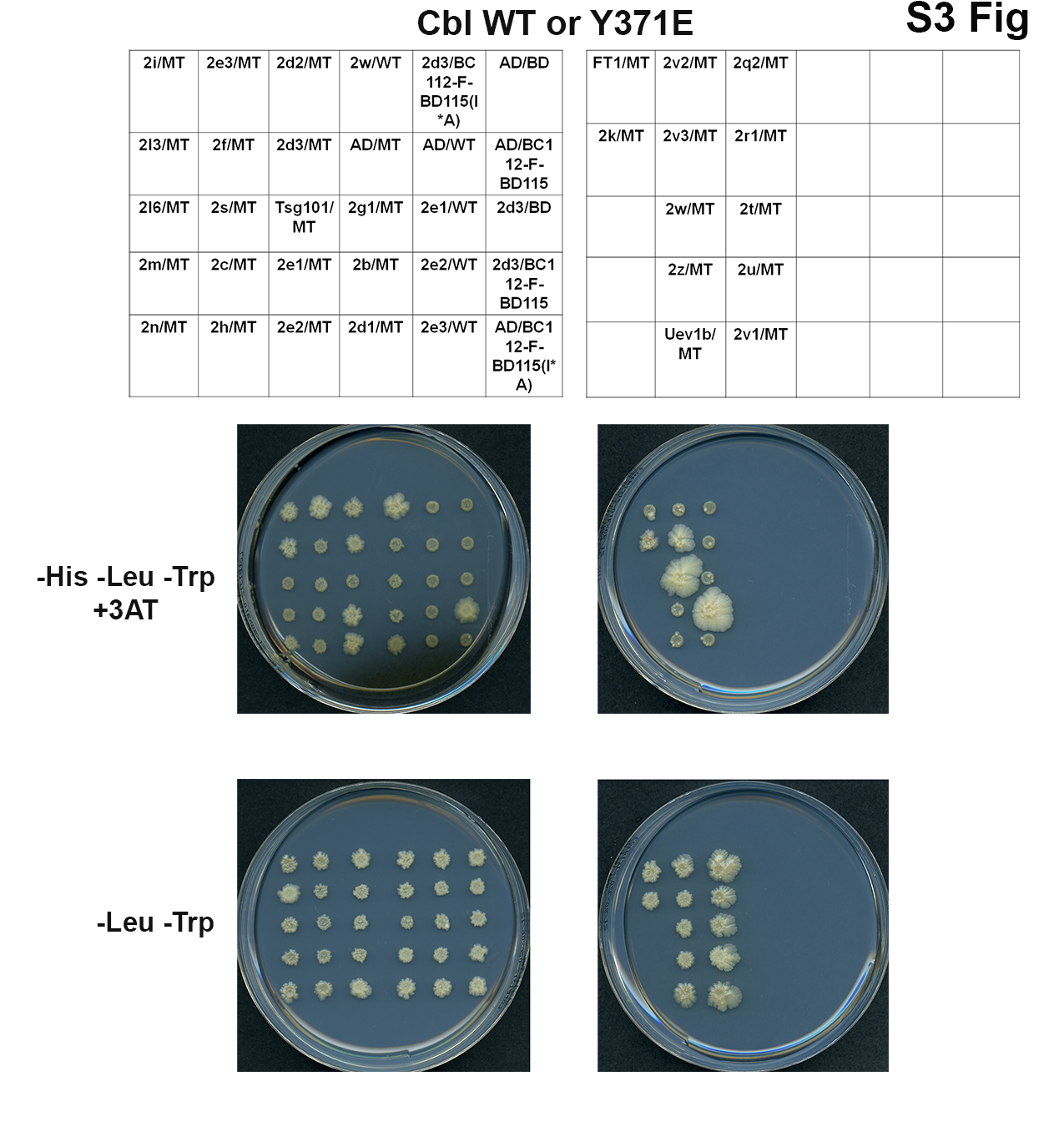

Supplement: S3 Fig — E2 screening with activated Cbl Y371E (MT) protein by yeast two-hybrid assay. Cbl Y371 expression plasmids were transformed together with each of 29 E2s plasmids into yeast. Transformed yeasts were plated on either -His, -Leu, -Trp, +3AT interaction selection medium (upper panels) or -Leu -Trp medium to confirm that the yeasts received both plasmids (lower panels). The key is shown above the plates. BC112-F-BD115 and BC112-F-BD115 I26A (I*A) with Ube2ds were used as positive and negative controls, respectively. (TIF) [file pone.0216967.s003.tif]

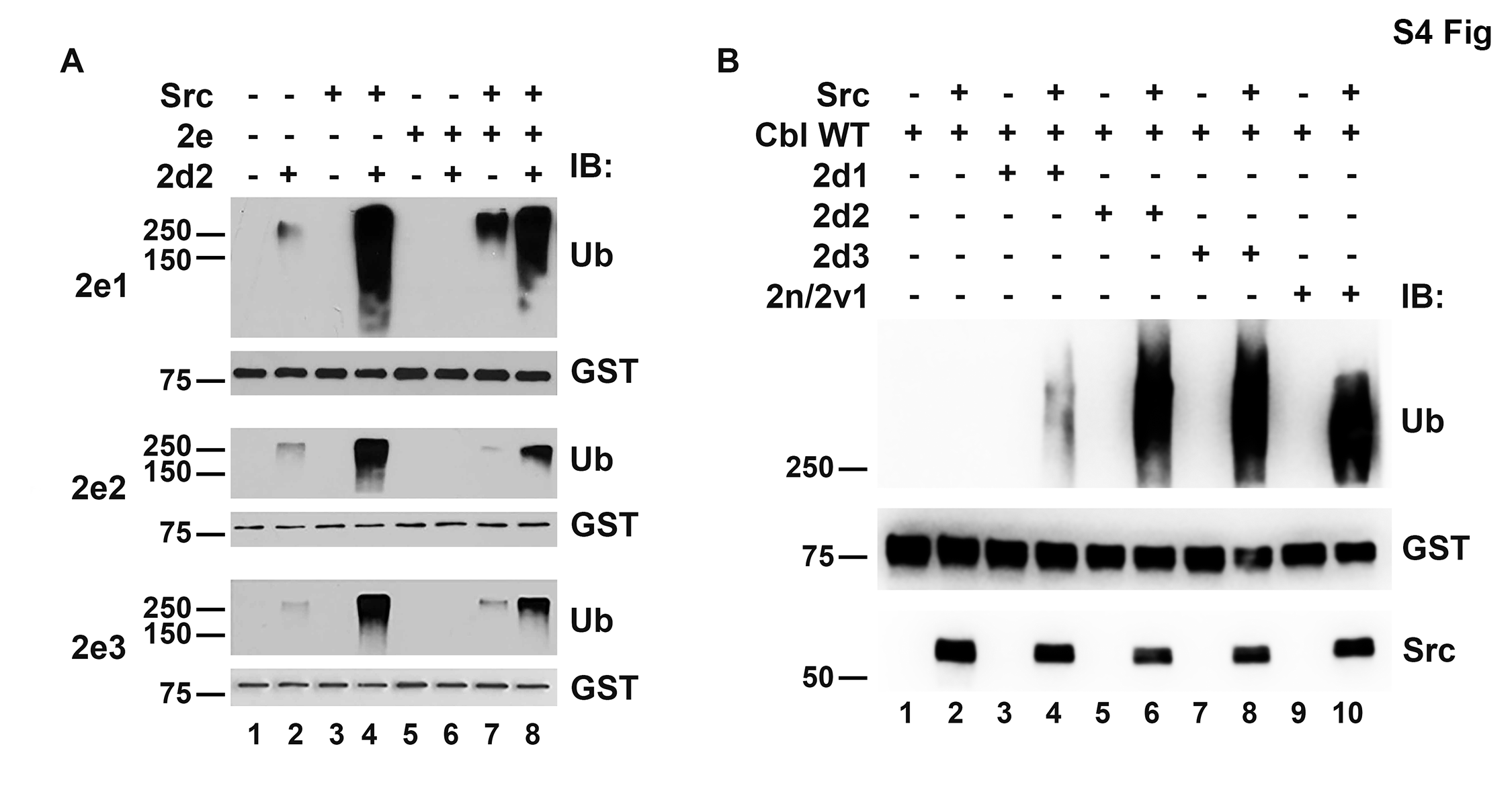

Supplement: S4 Fig — (A) In vitro E3 assays were performed with Ube2e1, Ube2e2 and Ube2e3 respectively in absence or presence of Ube2d2 or Src as indicated. (B) In vitro E3 assays were performed with Ube2d1, Ube2d2, Ube2d3, or Ube2n/2v1 in absence or presence of Src as indicated. Assays were performed with GST-Cbl for 60 min and then immunoblotted with antibodies against ubiquitin, Src, and GST. MW in kDa is shown to the left of the panels. (TIF) [file pone.0216967.s004.tif]

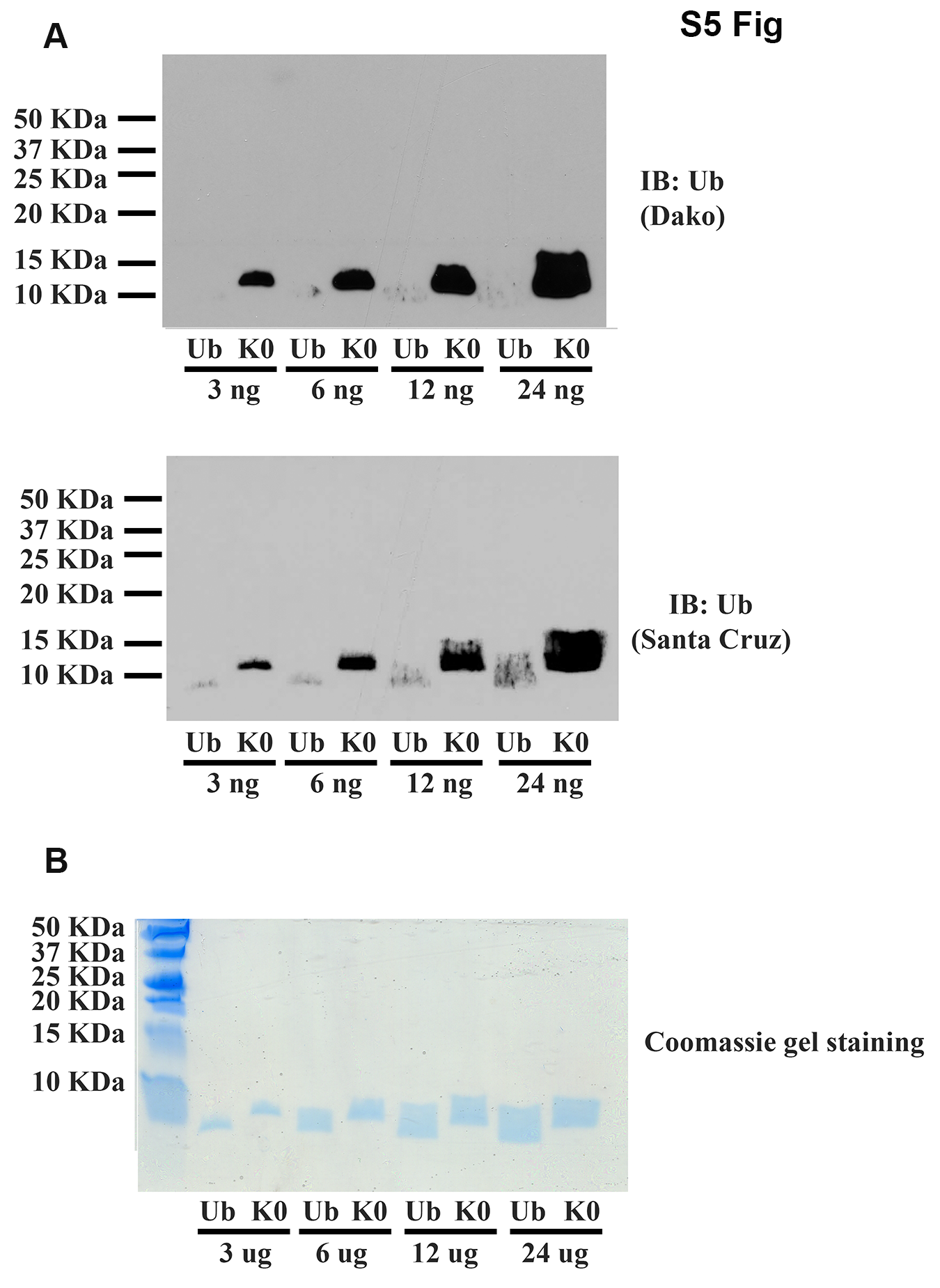

Supplement: S5 Fig — (A) Equal amounts of wildtype or K0 ubiquitin recombinant proteins were immunoblotted with the anti-ubiquitin antibodies P4D1 from Santa Cruz or Z0458 from Dako as indicated. (B) Equal amounts of wildtype or K0 ubiquitin recombinant proteins were separated by PAGE and then stained with Gelcode Blue Stain (ThermoFisher Scientific) to demonstrate equal loading. The immunoblots in A were direct 1000-fold dilutions of the samples run in B. (TIF) [file pone.0216967.s005.tif]

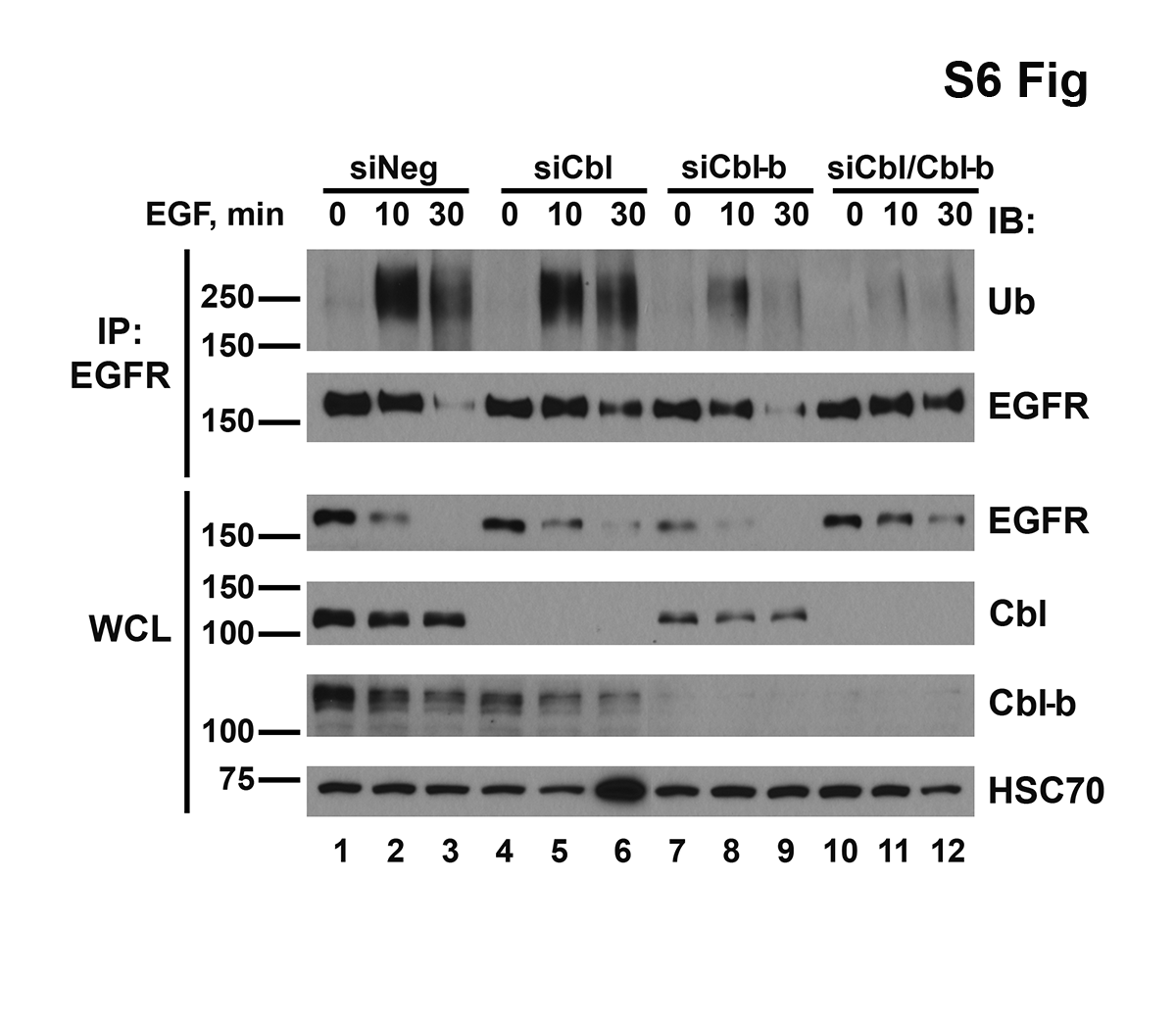

Supplement: S6 Fig — Hela cells were transfected with siRNA targeting Cbl, Cbl-b or both proteins for 48 hours. The cells were either left untreated (0) or treated with 100 ng/mL EGF for 10 or 30 minutes. EGFR was immunoprecipitated and analyzed for ubiquitination with anti-Ub antibodies. The efficient knockdown of each protein was demonstrated in whole cell lysates (WCL) by probing with anti-Cbl and anti-Cbl-b antibodies. MW in kDa is shown to the left of the panels. (TIF) [file pone.0216967.s006.tif]
